# Recruitment of release sites underlies chemical presynaptic potentiation at hippocampal mossy fiber boutons

**DOI:** 10.1371/journal.pbio.3001149

**Published:** 2021-06-21

**Authors:** Marta Orlando, Anton Dvorzhak, Felicitas Bruentgens, Marta Maglione, Benjamin R. Rost, Stephan J. Sigrist, Jörg Breustedt, Dietmar Schmitz

**Affiliations:** 1 Charité – Universitätsmedizin Berlin, corporate member of Freie Universität Berlin and Humboldt-Universität zu Berlin, Berlin, Germany; 2 NeuroCure Cluster of Excellence, Berlin, Germany; 3 Department of Biology, Chemistry, and Pharmacy, Freie Universität Berlin, Berlin, Germany; 4 German Center for Neurodegenerative Diseases, Berlin, Germany; 5 Bernstein Center for Computational Neuroscience Berlin, Berlin, Germany; 6 Einstein Center for Neurosciences Berlin, Berlin, Germany; 7 Max Delbrück Center for Molecular Medicine, Berlin, Germany; Thomas Jefferson University, UNITED STATES

## Abstract

Synaptic plasticity is a cellular model for learning and memory. However, the expression mechanisms underlying presynaptic forms of plasticity are not well understood. Here, we investigate functional and structural correlates of presynaptic potentiation at large hippocampal mossy fiber boutons induced by the adenylyl cyclase activator forskolin. We performed 2-photon imaging of the genetically encoded glutamate sensor iGlu_u_ that revealed an increase in the surface area used for glutamate release at potentiated terminals. Time-gated stimulated emission depletion microscopy revealed no change in the coupling distance between P/Q-type calcium channels and release sites mapped by Munc13-1 cluster position. Finally, by high-pressure freezing and transmission electron microscopy analysis, we found a fast remodeling of synaptic ultrastructure at potentiated boutons: Synaptic vesicles dispersed in the terminal and accumulated at the active zones, while active zone density and synaptic complexity increased. We suggest that these rapid and early structural rearrangements might enable long-term increase in synaptic strength.

## Abstract 2

This study uses several high-resolution imaging techniques to investigate the structural correlates of presynaptic potentiation at hippocampal mossy fiber boutons, observing an increase in release sites and in release synchronicity accompanied by synaptic vesicle dispersion in the terminal and accumulation at release sites, but no modulation of the distance between calcium channel and release sites.

## Introduction

The term synaptic plasticity describes the ability of synapses to change their strength and efficacy over time. Long-term forms of synaptic plasticity are postulated as cellular mechanisms responsible for learning and memory [1,2]. Changes in synaptic strength are paralleled by changes in the structure of neuronal contacts that underlie long-term circuit reorganization [3,4]. A long-term increase in synaptic strength (long-term potentiation [LTP]) can be expressed postsynaptically, importantly by changes in postsynaptic receptor number or properties [5], but also presynaptically, by changes in neurotransmitter release [4].

In this study we investigated chemical presynaptic potentiation at large hippocampal mossy fiber boutons (hMFBs) [6]. Dentate gyrus granule cells form excitatory synapses onto spines of proximal dendrites of CA3 pyramidal neurons [7]. hMFBs were the first synapses described to undergo a NMDA-receptor-independent form of LTP that is both induced and expressed at the presynaptic terminal [8,9]. Here, the increase in intracellular calcium following high-frequency firing activates calcium/calmodulin-dependent adenylyl cyclases, which leads to an increase in the intracellular concentration of cyclic adenosine monophosphate (cAMP), which, in turn, drives the activation of protein kinase A (PKA). Ultimately, PKA phosphorylation events result in a long-lasting increase in neurotransmission [6,10].

A variety of knockout models provided information on potential PKA phosphorylation targets required for presynaptic potentiation. Rab3A [11], its interaction partners RIM1α and Munc13 [12], and synaptotagmin-12 [13] have all been shown to be crucial for presynaptic LTP at hMFBs. A-kinase anchoring protein 7 (AKAP7) localizes PKA presynaptically at hMFBs and is required for cAMP-induced LTP and pattern separation behaviors [14]. Despite this body of literature, it is still not known exactly how these proteins are involved in presynaptic LTP induction and expression [4].

Presynaptic LTP at hMFBs has traditionally been described as the long-lasting increase in release probability (*P*_r_) [9,15,16], but vesicle availability as well as changes in the number of release sites could also play a major role in setting the stage for increased neurotransmission. Indeed, at hMFBs, an increase in docked vesicles has been proposed as a mechanism for post-tetanic-potentiation [17]. At cerebellar parallel and climbing fiber synapses, PKA and its vesicle-associated target, synapsin, dynamically control release site occupancy and dictate the number of vesicles released per action potential without altering *P*_r_ [18]. Moreover, activation of silent synapses and addition of release sites have been suggested as potential mechanisms for the expression of presynaptic LTP at hMFBs [19,20]. Changes in the number and localization of docked vesicles [21], potentially accompanied by addition of new release sites, could underlie functional changes at hMFBs.

The morphological complexity of mossy fiber boutons has been shown to increase in mice kept in an enriched environment [22] and in cryo-fixed organotypic slices treated with the potassium channel blocker tetraethylammonium [23]. Moreover, the transport of active zone (AZ) proteins via vesicular cargo to nascent AZs likely underlies long-term plasticity in the hippocampus [24].

Synapses are organized in nanocolumns [25]. Stimulated emission depletion (STED) microscopy allowed the detection of a rapid reorganization of pre- and postsynaptic modules upon chemically induced structural plasticity [26]. Changes in AZ nano-architecture upon LTP induction have also been hypothesized to sustain the increase in *P*_r_. Direct double patch-clamp experiments from presynaptic hMFBs and postsynaptic CA3 pyramidal neurons indicated a relatively long distance (70 to 80 nm) between calcium channels and synaptic vesicles (SVs) and therefore a functionally “loose coupling” between calcium source and calcium sensor [27]. Loose coupling is responsible for the intrinsically low *P*_r_ of this synapse [28]. Remarkably, experiments at dissociated hMFBs suggested an induced decrease in coupling distance between calcium channels and calcium sensor as a possible mechanism for LTP expression [29].

The complexity of the phenomenon and the fact that a variety of different experimental models have been used in the past decades might explain why we currently face several diverging theories to explain hMFB presynaptic LTP.

Our aim, in this context, was to characterize the ultrastructural and functional correlates of chemical presynaptic potentiation in brain slices to clarify whether and how synapses, vesicles, or AZs reorganize to express and sustain the long-term increase in neurotransmitter release. By means of 2-photon fluorescent imaging of glutamate release, STED microscopy, and 3D transmission electron microscopy (EM) analysis, we addressed the following questions: Do the addition of release sites and the rearrangement of AZ nano-architecture play a role in presynaptic potentiation? How does glutamate release dynamics change upon presynaptic potentiation?

## Results

### Increased presynaptic surface area of transmitter release at potentiated mossy fibers

**Fig 1:**
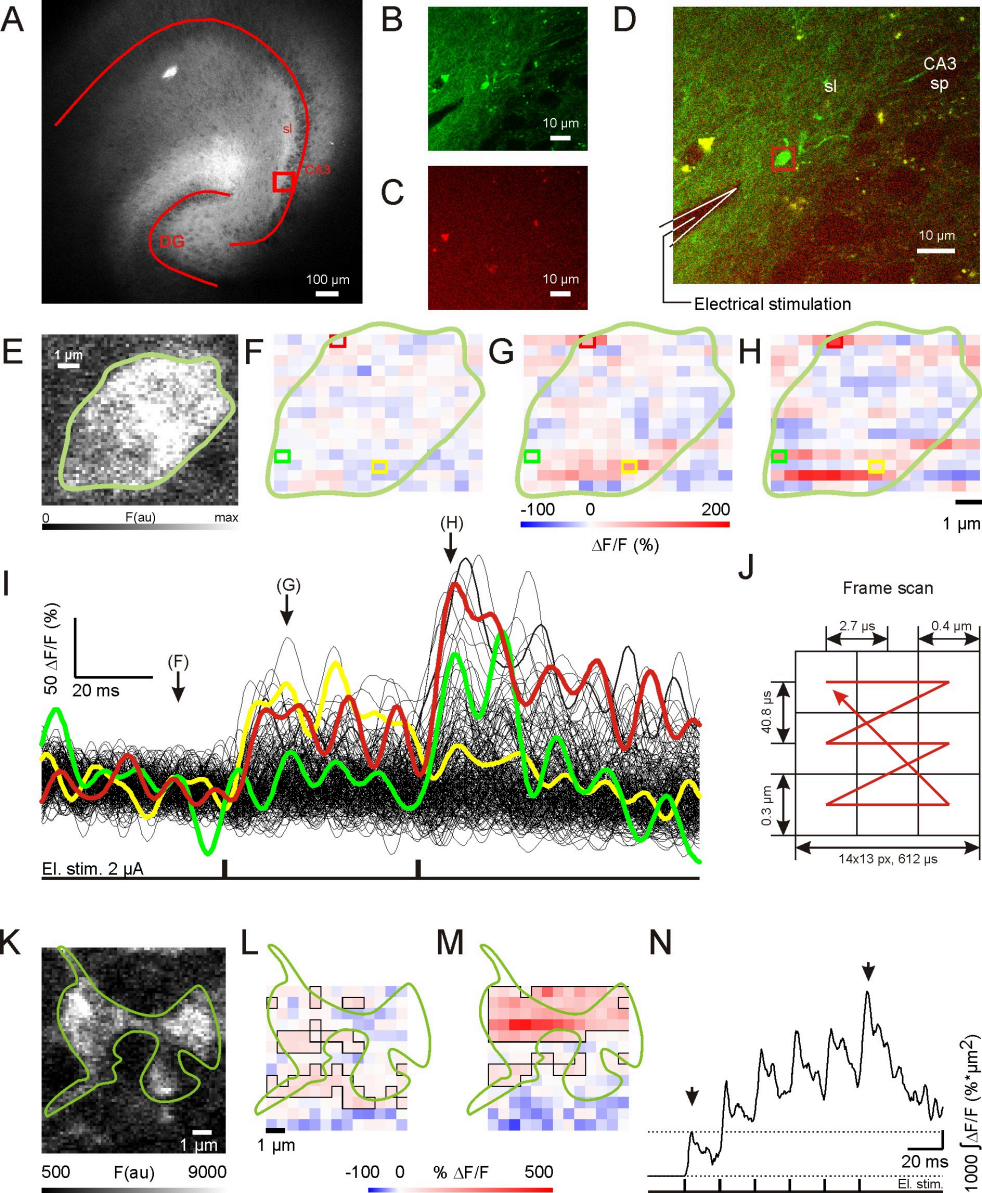
Two-photon imaging of single hippocampal mossy fiber bouton (hMFB) iGlu_u_ transients. (A) Fluorescent image of an organotypic hippocampal slice culture 3 wk after transfection with the genetically encoded glutamate sensor iGlu_u_ in dentate gyrus (DG) granule cells. The square shows the region in (B–D). DG and CA3 are outlined by overlay. sl, stratum lucidum. (B) iGlu_u_ fluorescent signal acquired by 2-photon imaging in stratum lucidum (average of 15 frames). (C) Image of nonspecific autofluorescence with emission > 600 nm. (D) Composite of (B) and (C). The red rectangle marks the recorded area of the hMFB shown in (E). Note the position of the stimulation electrode indicated by the drawing. sp, stratum pyramidale. (E) High-resolution image illustrating the hMFB shown in (B) and (D) immediately before the high-speed imaging recordings. A hand-drawn green curve contours the bouton. (F–H) Single frames of the same hMFB showing Δ*F/F* signals at rest (F) and at the peak response after the first (G) and second (H) electrical stimulation in control conditions. The green line in (F–H) contours the hMFB silhouette shown in (E). Colored boxes represent pixels for which the intensity plots are shown in (I). (I) Plot representing dynamic Δ*F/F* fluorescent signals for each pixel in (F–H). Colored traces represent signals from pixels in colored boxes in (F–H). (J) Scheme illustrating the 2-photon laser scanning pattern with mean spatiotemporal resolution characteristics. (K) High-resolution image of a hMFB used for high-frequency stimulation experiments. The bouton contour was manually outlined by a green line. (L and M) Single frames of the hMFB illustrated in (K) showing Δ*F/F* signals at the peak response after the first (L) and sixth (M) electrical stimulation in control conditions. The green line shows the hMFB location. Contoured pixels represent active area. Note elevated pixel intensity and active area after 50-Hz stimulation. (N) The trace represents cumulative response dynamics for the given hMFB example in (K–M). The arrows indicate time points taken for illustration in (L) and (M). Note the almost 4-fold increase in cumulative Δ*F/F* signal after the last stimulation compared to the first, which illustrates the operating range of the iGlu_u_ sensor. El. stim., electrical stimulation. The data underlying this figure can be found at doi: 10.5281/zenodo.4498214.

To investigate neurotransmission dynamics, we monitored glutamate release in the stratum lucidum of CA3 (Fig 1A), a region close to CA3 pyramidal cell bodies, where hMFBs form synapses on proximal dendritic spines of CA3 pyramidal neurons. We imaged glutamate release from hMFBs by 2-photon microscopy, using the genetically encoded and plasma-membrane-bound glutamate sensor iGlu_u_ with low sensitivity (Kd = 600 μM) [30] (Fig 1A, 1B, 1D and 1G).

iGlu_u_ expression did not alter basic neurotransmission (S2 Fig), and its specific fluorescent signal was readily distinguishable from background autofluorescence (Fig 1C; overlaid to iGlu_u_ signal in Fig 1D). Electrical stimulation of granule cell axons elevated iGlu_u_ fluorescence intensity in a complex spatiotemporal pattern (Fig 1E–1I). To assess the dynamic characteristics of iGlu_u_ transients, the area occupied by suprathreshold pixels (active area) and the cumulative, mean, and maximal amplitudes of Δ*F/F* signals for pixels in the active area were analyzed (see Methods for details). We tested the range of iGlu_u_ sensor response by monitoring the cumulative amplitude of single-hMFB iGlu_u_ dynamics after a train of 6 stimuli at 50 Hz (Fig 1K–1N). When we compared the iGlu_u_ signal of the last stimulus (Fig 1M) to that of the first stimulus (Fig 1L), we could monitor a 4-fold increase in the cumulative amplitude (Fig 1N).

**Fig 2:**
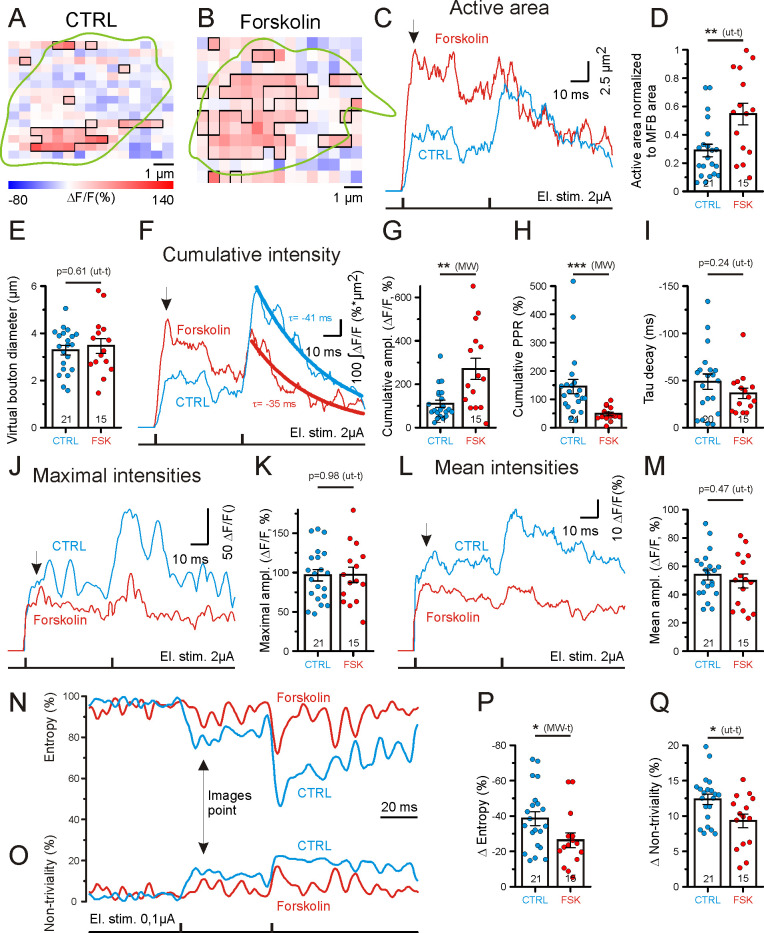
Forskolin increases the presynaptic surface area of glutamate release and the spatial synchronization of glutamate release within hMFBs. (A and B) Example images illustrating the spatial distribution of Δ*F/F* signals for 2 different hMFBs in control conditions (A) and in the presence of forskolin (B). The manually drawn green curve contours the profile of the recorded hMFB, based on the high-resolution image acquired before the stimulation experiment. The Δ*F/F* signals are taken at the peak responses to a first electrical stimulation (the time point is indicated by a black arrow in (C, F, J, L, N, and O). Suprathreshold pixels (pixels with Δ*F/F* intensities more than 3 × SD of the baseline signal, i.e., 50 ms before the stimulation) are contoured with a black line and represent the active area. Note the larger fraction of red pixels in the presence of forskolin (B) at equal intensities. (C) Example traces representing active area (the area of suprathreshold pixels) dynamics for hMFBs under control conditions (blue) and in the presence of forskolin (red). (D) Bar graph showing the active area at peak of response to the first stimulation normalized to the hMFB area. Note forskolin-mediated increase of hMFB active area. (E) Forskolin does not change the virtual bouton diameter (diameter of a circle with area equal to the area of the recorded bouton) of hMFBs. The bouton area was calculated using high-resolution images obtained before high-speed recordings. (F) Traces of cumulative intensities (spatial integral of suprathreshold pixels). The signal decay after the second stimulation is fitted with a monoexponential curve (thick lines) to identify the tau of decay (τ). (G–I). Bar graphs indicating the significant increase in cumulative amplitude in the presence of forskolin (maximal response to the first stimulation) (G), the decrease in the cumulative PPR (H), and the unchanged tau of decay of the cumulative intensities (I). (J) Traces of maximal Δ*F/F* values for suprathreshold pixels. (K) Bar graph showing that forskolin does not affect the maximal amplitude. (L) Traces of mean Δ*F/F* for suprathreshold pixels. (M) Bar graph showing that forskolin does not affect the mean amplitude. (N and O) Example traces representing informational entropy (N) and non-triviality (O) (see definitions in Methods) calculated for 2D patterns of Δ*F/F* spatial distributions at each time point for different hMFBs under control conditions (blue traces) and in the presence of forskolin (red traces). (P and Q) Bar graphs showing significantly decreased amplitudes of entropy (P) and non-triviality (Q) at the peak response to the fist stimulation. ampl., amplitude; CTRL, control; El. stim., electrical stimulation; FSK, forskolin; hMFB, hippocampal mossy fiber bouton; MFB, hippocampal mossy fiber bouton; MW, Mann–Whitney *U* test; MW-t, Mann–Whitney *U* test; PPR, paired-pulse ratio; ut-t, unpaired *t* test. The data underlying this figure can be found at doi: 10.5281/zenodo.4498214.

Presynaptic potentiation at hMFBs was induced by incubating organotypic hippocampal cultures for 15 min in 50 μM forskolin. Forskolin-treated hMFBs, in comparison with untreated hMFBs, showed a significant increase in the fraction of active area (Fig 2A–2D), whereas forskolin did not change the virtual bouton diameter (diameter of a circle with an area equal to the area of the recorded bouton) (Fig 2E). Forskolin also significantly elevated the cumulative amplitude of iGlu_u_ transients (Fig 2F and 2G). The average paired-pulse ratio (PPR) of the cumulative amplitudes (PPR_Cum_) under control conditions was 1.45 ± 0.25 (Fig 2H), a value that is close to the PPR for excitatory postsynaptic currents (EPSCs) recorded at 2 mM extracellular Ca^2+^ [31]. The cumulative amplitude reflects the total amount of released glutamate [32,33] and is negatively correlated with PPR (S1A Fig), thus showing an activity-dependent form of short-term plasticity. Neither the mean amplitude nor the active area correlated with PPR (S1B and S1C Fig). Taken together, these data indicate that the cumulative amplitude of iGlu_u_ transients is the parameter best suited for a comparison with evoked EPSCs.

hMFBs in forskolin-treated slices showed a significant decrease in PPR_Cum_ (Fig 2F and 2H). This is in accordance with the potentiation effect of forskolin on hippocampal mossy fiber transmission, which has been extensively characterized by electrophysiological recordings [34,35].

The measured active area depends on several factors, such as the number of active release sites, the concentration of glutamate in the synaptic cleft, the bouton size, and the effectiveness of glutamate clearance. To target the last of these, we analyzed the decay kinetics of the cumulative iGlu_u_ transient by fitting a monoexponential decay function to the signal, and observed similar decay kinetics for control and potentiated boutons (Fig 2F and 2I). Of note, the size of the active area correlated with the cumulative amplitude (S1D Fig), but not with the mean (S1E Fig) or maximal amplitudes (S1F Fig). The maximal (Fig 2J and 2K) and mean (Fig 2L and 2M) amplitudes of the iGlu_u_ signal in the population of active pixels were not significantly altered by forskolin (Fig 2J–2M), indicating that the concentration of glutamate released in the synaptic cleft does not contribute to forskolin-induced potentiation at hMFBs. However, this result may also be explained by glutamate sensor saturation. To test this, we stimulated mossy fibers with 5 pulses at 50 Hz (S2A–S2E Fig). We saw that single pulse responses were located in the lower part of the iGlu_u_ working range (Figs 2J, 2K and S2E). The absolute fluorescence of hMFBs at rest did not differ significantly between control and forskolin-treated groups (S2F Fig), ruling out a role of different iGlu_u_ expression in mediating the observed effects on active area and mean or maximal amplitudes. Moreover, iGlu_u_ imaging of the same hMFBs before and after forskolin treatment (S3 Fig) also revealed a significant elevation of active area without a change in mean amplitude (S3B, S3D and S3E Fig). However, the interpretation of these results was complicated by iGlu_u_ bleaching. (S3A and S3C Fig). Taken together, our results indicate that, at hMFBs, the forskolin-induced increase of active area likely reflects an increase in the area of active glutamate release, rather than a diffusional glutamate spread.

Thus, we show that forskolin potentiates presynaptic glutamate release at hMFBs by increasing the presynaptic membrane area at which exocytosis occurs.

### Enhancement of release synchronicity

As shown here and previously [36], different iGlu_u_ hotspots can display opposite paired-pulse behaviors and are activated in an apparently stochastic manner (Fig 1E–1H and 1K). This means that hMFBs likely harbor a probabilistic fraction of silent release sites, which may be activated after forskolin treatment [19,20]. Unfortunately, diffraction-limited light microscopy does not allow us to directly visualize glutamate release from single release sites. However, we can indirectly assess the fraction of silent release sites by the spatial randomness and anisotropy of iGlu_u_ transients. It can be assumed that a spatially inhomogeneous distribution of iGlu_u_ transients reflects a large number of silent release sites, while a homogeneous distribution of the iGlu_u_ signal indicates a smaller fraction of silent release sites. To test if forskolin would increase the number of active release sites, we analyzed the informational entropy and non-triviality spatial patterns of the iGlu_u_ transients [37]. Before electrical stimulation, hMFBs had a random Δ*F/F* spatial pattern with a maximal entropy and minimal non-triviality (Figs 1F, 2N and 2O). The evoked glutamate release from hMFB resulted in an increase in iGlu_u_ fluorescence on presynaptic membrane portions that are closest to the release sites. This rendered the profile of Δ*F/F* a heterogeneous and anisotropic presynaptic landscape, i.e., it decreased the entropy and increased the non-triviality of the Δ*F/F* spatial pattern (Fig 2N and 2O). Forskolin-treated hMFBs showed significantly smaller changes of entropy (Fig 2N and 2P) and non-triviality (Fig 2O and 2Q) than untreated boutons. In other words, forskolin increases the spatial homogeneity and isotropy of iGlu_u_ transients in hMFBs. For this analysis, we used the area of the whole synaptic bouton and even some small portion of the surrounding space. This means that forskolin effects on entropy and non-triviality may be associated with the increased fraction of pixels whose signal is changed by glutamate release rather than by individual pixel behavior. However, neither entropy nor non-triviality correlated with the size of the active area (S1G Fig).

The amount of released glutamate might also affect entropy and non-triviality. However, neither mean nor cumulative amplitudes correlated with entropy and non-triviality (S1H and S1I Fig).

Together, our data likely indicate that forskolin increases the portion of simultaneous release events.

### No change in coupling distance at potentiated synapses

The increase in releasing area at potentiated hMFBs could be driven by addition of new release sites or by activation of functionally silent release sites. Since hMFBs exhibit loose coupling between calcium channels and primed vesicles [27], such activation could be the result of a tightening of the coupling distance [29]. This could also explain the increase in glutamate release synchrony between multiple release sites, as a tighter coupling would drive vesicle fusion more reliably [38].

**Fig 3:**
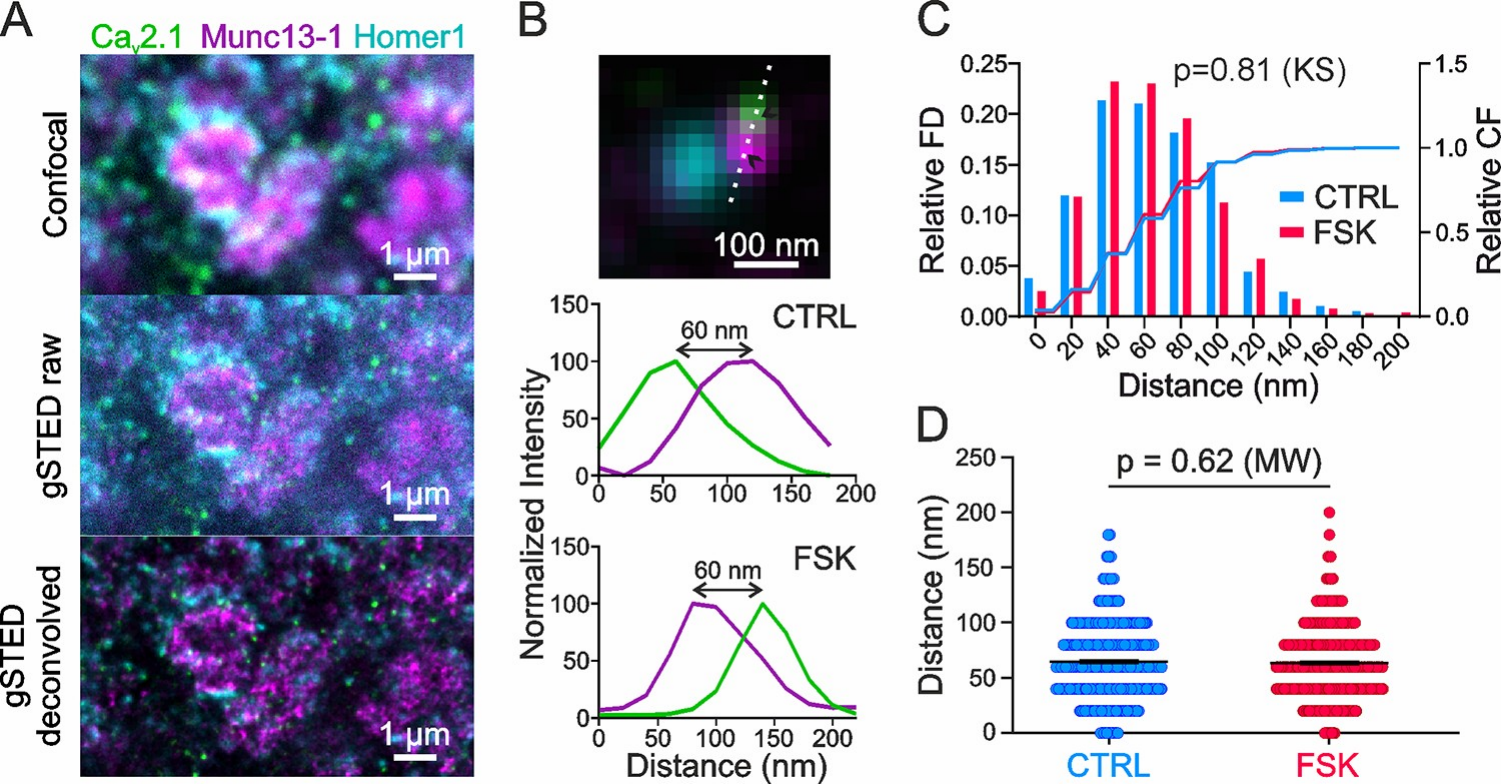
Coupling distance between Cav2.1 and Munc13-1 in the CA3 stratum lucidum is unchanged in control versus forskolin. (A) Example scan in ZnT3-positive area of the CA3 stratum lucidum in 100-μm-thick hippocampal slices: confocal scan (top), raw gSTED scan (middle), and deconvolved gSTED scan (bottom). Staining for Cav2.1 (green), Munc13-1 (magenta), and Homer1 (cyan). (B) Example of an analyzed synapse: The distance between Cav2.1 (green) and Munc13-1 (magenta) was measured only if they were in close proximity to a Homer1-positive spot (cyan). Line profiles were plotted at the dotted line (top), drawn through the intensity maxima of the Cav2.1 and Munc13-1 signals (arrowheads). The distance was calculated between the intensity maxima of the Cav2.1 and Munc13-1 signals, shown in the example normalized intensity plots for control (middle) and forskolin-treated (bottom). (C) The distribution of measured distances between Cav2.1 and Munc13-1 in the CA3 stratum lucidum is unchanged in control versus forskolin-treated (*p =* 0.81, 2-sample Kolmogorov–Smirnov test). Frequency distribution (left *y*-axis, bars) and cumulative frequency (right *y*-axis, lines) with a bin size of 20 nm, for control (blue) and forskolin-treated (red). (D) The mean distance between Cav2.1 and Munc13-1 in the CA3 stratum lucidum is unchanged in control versus forskolin-treated. Scatter plot from all measured synapses: distances (nm) for CA3 control in blue (*n* = 584 synapses from 11 animals) and CA3 forskolin-treated in red (*n* = 525 synapses from 11 animals). Bar graphs show mean values ± SEM. Significance tested with Mann–Whitney *U* test (*p =* 0.62). CS, cumulative frequency; CTRL, control; FD, frequency distribution; FSK, forskolin; gSTED, time-gated stimulated emission depletion; KS, Kolmogorov–Smirnov test; MW, Mann–Whitney *U* test. The data underlying this figure can be found at doi: 10.5281/zenodo.4498214.

To determine whether a change in the distance between calcium source and release sites contributes to the increase in neurotransmitter release during presynaptic potentiation at hMFBs, we performed time-gated STED (gSTED) microscopy on forskolin-treated and untreated acute brain slices obtained from the same animal. Slices were stained for Cav2.1, to detect P/Q-type calcium channels; for Munc13-1, as a marker for release sites [39]; and for Homer1, a postsynaptic marker for glutamatergic synapses (Fig 3A). The CA3 stratum lucidum was identified by staining for a mossy-fiber-specific zinc transporter (ZnT3; S4E Fig). gSTED allowed us to detect a punctate immunostaining of synaptic proteins. Here, we refer to these puncta as clusters, as in a previous study [40]. We measured the distance between presynaptic Cav2.1 and Munc13-1 clusters only when they were juxtaposed to a Homer1 cluster, making sure that the clusters belonged to the same AZ (Fig 3B).

The distances measured between Cav2.1 and Munc13-1 clusters were unchanged between control and potentiated slices. Measured distances ranged up to 180 nm for controls (*n* = 584 synapses from 11 animals) and up to 200 nm for forskolin-treated synapses (*n* = 525 synapses from 11 animals). Ninety-five percent of distances were shorter than 120 nm (Fig 3C). The mean distance was 64.62 ± 1.43 nm for control and 63.62 ± 1.43 nm for forskolin-treated slices (Fig 3D; *p =* 0.62, Mann–Whitney *U* test), when comparing all measured synapses. The same result was reflected in the means of the animals: The mean distance was 64.21 ± 3.63 nm for control and 63.54 ± 2.93 nm for forskolin-treated slices. The measured mean distance is consistent with the loose coupling configuration of hMFBs previously determined by electrophysiological recordings [27], as well as by a previous study measuring coupling distances by gSTED in situ [40].

To validate that our gSTED analysis could indeed retrieve shorter coupling distances, we measured coupling distances in the CA1 stratum radiatum of untreated slices (*n* = 491 synapses from 11 animals; S4A–S4D Fig). Here, our method revealed a significantly shorter distance between Cav2.1 and Munc13-1 clusters than observed in control synapses in the CA3 stratum lucidum: The mean distance for CA1 synapses was 54.87 ± 1.47 nm in contrast to 64.62 ± 1.43 nm for CA3 control synapses (*p* < 0.0001, Mann–Whitney *U* test). Also, the mean distances per animal showed the same result, with a mean of 54.79 ± 2.56 nm for CA1 compared with 64.21 ± 3.63 nm for CA3 control slices (*p =* 0.047, unpaired *t* test). Importantly, the frequency distribution was shifted towards smaller values in CA1, with most measured distances being between 20 and 60 nm (S4C Fig), in line with distance simulations for Schaffer collateral synapses [41].

Taken together, our gSTED measurements indicate that modulation of coupling distances upon presynaptic potentiation at hMFBs is highly unlikely, suggesting that other mechanisms account for the increase in neurotransmitter release after potentiation, such as the insertion of new calcium channels and/or new release sites.

### Increased presynaptic complexity and AZ density after forskolin treatment

**Fig 4:**
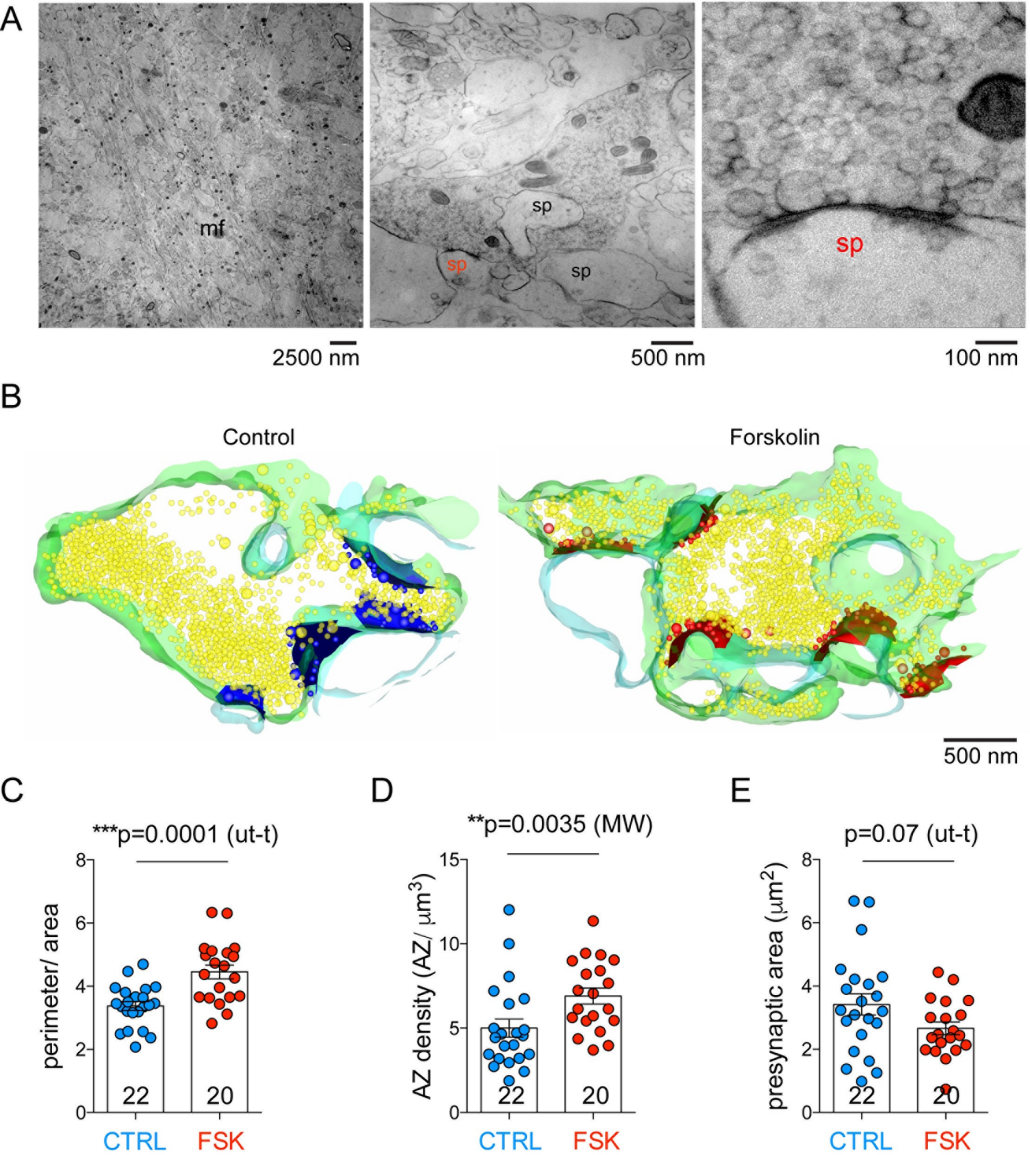
Three-dimensional EM analysis reveals an increase in presynaptic complexity and AZ density in forskolin-treated cryo-fixed acute slices. (A) EM image of the stratum lucidum of the hippocampal CA3 region. Mossy fiber axon bundles (mf) are visible in the left panel. In the central panel, large presynaptic terminals contacting multiple spine heads (sp) are visible. The right panel shows a high-magnification image of a single AZ; this is a magnification of the spine marked in red (sp) in the middle panel. (B) Partial 3D reconstruction computed from manually segmented serial images of hMFBs in control conditions or after forskolin treatment. Presynaptic membrane is green, postsynaptic membrane is light blue, synaptic vesicles are yellow, AZs and docked or tethered vesicles are blue (control) or red (forskolin). (C) Bar graph indicating the quantification of bouton complexity (perimeter/area) obtained from images like the middle image of (A); bouton complexity was larger in forskolin-treated terminals (*p =* 0.0001, unpaired *t* test). (D) Bar graph indicating the quantification of AZ density (AZ/μm^3^) obtained from 3D reconstructions like those in (B); AZ density was larger in forskolin-treated terminals (*p =* 0.0035, Mann–Whitney *U* test). (E) Bar graph indicating the quantification of presynaptic area (μm^2^) obtained from images like the middle image of (A); presynaptic area was unchanged in forskolin-treated terminals when compared to controls (*p =* 0.07, unpaired *t* test). In all graphs, scatter points indicate individual boutons, *n* = 22 boutons for control and 20 boutons for forskolin-treated slices from 4 animals. Values represent mean ± SEM. AZ, active zone; CTRL, control; EM, electron microscopy; FSK, forskolin; hMFB, hippocampal mossy fiber bouton; MW, Mann–Whitney *U* test; ut-t, unpaired *t* test. The data underlying this figure can be found at doi: 10.5281/zenodo.4498214.

To investigate the close-to-native ultrastructure of hMFBs with a nanometer resolution we used rapid high-pressure-freezing (HPF) and EM imaging of acute slices (Fig 4A). hMFBs were easily identifiable for their size and the fact that they make contact onto multiple spine heads [42] (Figs 4A and S5A) in the stratum lucidum of the CA3 region of the hippocampus (Figs 4A and S5A). Presynaptic potentiation was induced by incubating acute slices for 15 min in 50 μM forskolin. After HPF, the ultrastructure of potentiated hMFBs was compared to that of control hMFBs from the same mouse. Forskolin treatment increased synaptic complexity (measured as the perimeter of the whole presynaptic bouton divided by the bouton area in 2D images) (Fig 4C). To test the hypothesis that the activation of silent presynaptic release sites might contribute to chemical presynaptic potentiation at hMFBs [19,20], we analyzed the density of AZs in partial 3D reconstructions. In forskolin-treated terminals, we observed an increase in AZ density, measured as AZ number per cubic micron (Fig 4D). The presynaptic area measured in 2D profiles of hMFBs was not significantly altered (Fig 4E), although we observed a tendency towards a reduced presynaptic area under forskolin treatment, probably due to the increase in presynaptic complexity. In a set of parallel experiments, we investigated the ultrastructure of hMFBs in acute sagittal slices after chemical fixation (S5 Fig). In this preparation, forskolin treatment increased hMFB AZ density (S5D Fig), while synaptic complexity and presynaptic area were unchanged (S5B and S5C Fig).

### SVs disperse upon potentiation

**Fig 5:**
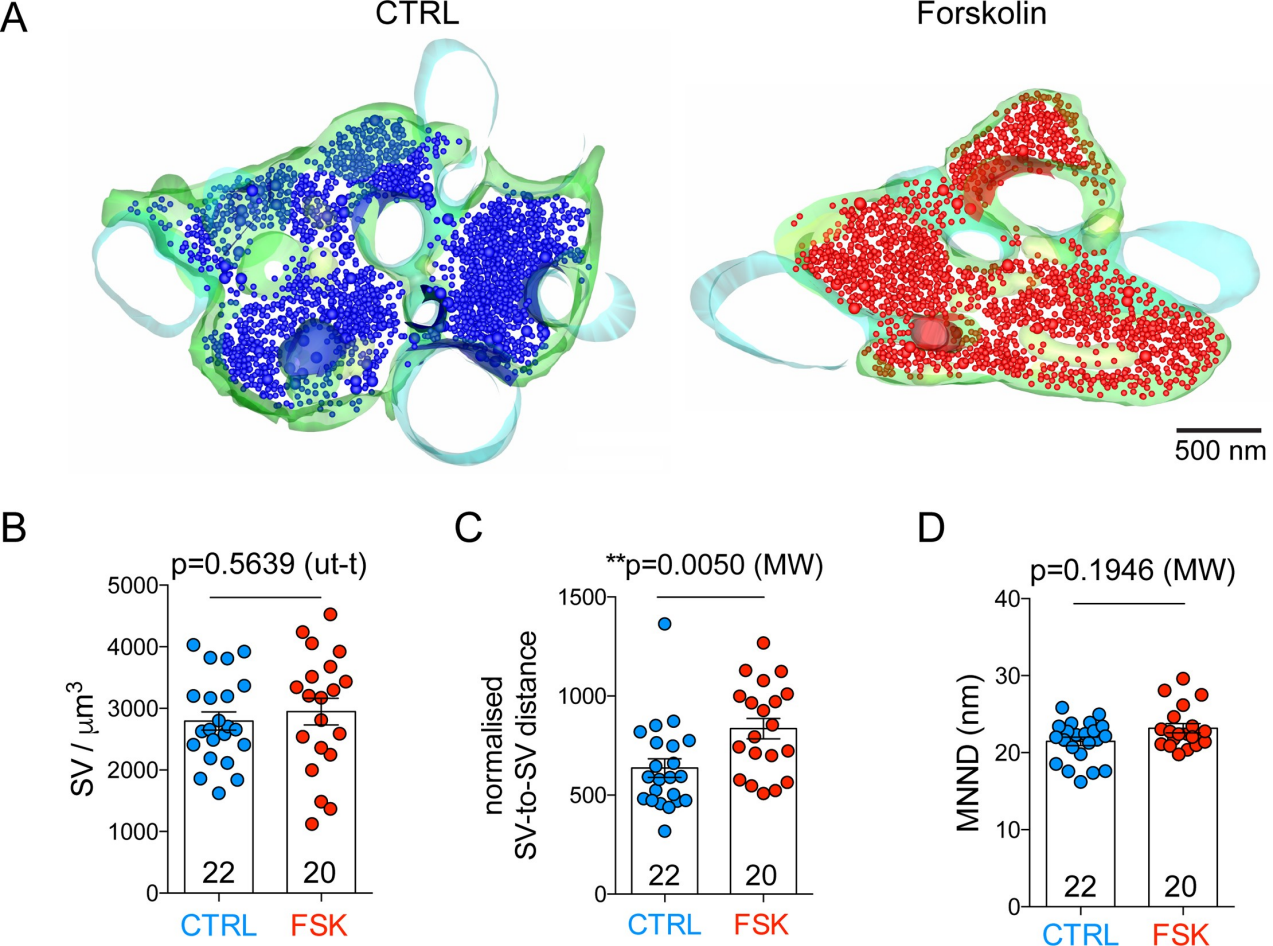
SVs disperse upon forskolin-induced presynaptic potentiation in cryo-fixed acute slices. (A) Partial 3D reconstruction of hippocampal mossy fiber boutons in control conditions or after forskolin treatment. Presynaptic membrane is green, postsynaptic membrane is light blue, and SVs are blue (control) or red (forskolin). (B) Bar graphs indicating the quantification of SV density per cubic micron of reconstructed volume; SV density was comparable in forskolin-treated and control terminals (*p =* 0.5639, unpaired *t* test). (C) Bar graphs indicating the quantification of SV distance from other SVs normalized by the volume of the reconstruction (nm/μm^3^); distance between vesicles was increased in forskolin-treated terminals (*p =* 0.0050, Mann–Whitney *U* test). (D) Bar graphs indicating the quantification of MNND between vesicles (nm); MNND was comparable in forskolin-treated and control terminals (*p =* 0.1946, Mann–Whitney *U* test). In all graphs, scatter points indicate individual boutons, *n* = 22 boutons for control and 20 boutons for forskolin-treated slices from 4 animals. Values represent mean ± SEM. CTRL, control; FSK, forskolin; MNND, mean nearest neighbor distance; MW, Mann–Whitney *U* test; SV, synaptic vesicle; ut-t, unpaired *t* test. The data underlying this figure can be found at doi: 10.5281/zenodo.4498214.

Forskolin-driven increase in cAMP concentration and the subsequent activation of PKA have recently been shown to act on synapsin to modulate short-term plasticity [43], multivesicular release [18], and vesicle availability [44]. We analyzed the 3D distribution of SVs in the presynaptic mossy fiber bouton and compared the number and localization of SVs under forskolin and control conditions. Forskolin did not provoke a change in SV density (Fig 5B); however, it induced SV dispersion inside the terminal. Thus, we measured the distance from each vesicle to all other vesicles in 3D and normalized it by the stack volume. In forskolin-treated hMFBs this distance was significantly increased (636.3 ± 47.26 for control and 836 ± 51.26 for forskolin-treated, *p =* 0.0050, Mann–Whitney *U* test; Fig 5C). We also measured the mean nearest neighbor distance (MNND) between vesicles. Since the size of our sections (*z*) is bigger than the nearest neighbor distance, we measured this parameter in 2D images. We found no significant difference in MNND between forskolin-treated and control synapses (Fig 5D). In chemically fixed slices, the increase in vesicle-to-vesicle distance after forskolin treatment was similar to that observed in cryo-fixed slices (S6C Fig), while SV density and MNND were unchanged (S6B and S6D Fig). Note that, due to the fact that the slice thickness is comparable to 2 times a vesicle diameter, the projection of vesicles in the *z*-dimension might result in a slight underestimation of the MNND.

Mitochondria are the most voluminous organelles in presynaptic terminals; hence, the difference in SV distribution might be a consequence of different mitochondrion volume in control and potentiated boutons. Mitochondria also have important functional relevance: They provide ATP, maintain calcium homeostasis in presynaptic terminals, and are thought to regulate SV mobility during plasticity [45]. For these reasons, we measured the volume of mitochondria as a percentage of the total volume of the reconstructed presynaptic terminal. No difference was found between control and potentiated synapses (data available at doi: 10.5281/zenodo.4498214). In summary, we found that forskolin treatment triggers the dispersion of SV in the hMFB, an effect that likely increases SV availability at the release sites.

### Increase in docked and tethered vesicle density upon forskolin-induced potentiation

**Fig 6:**
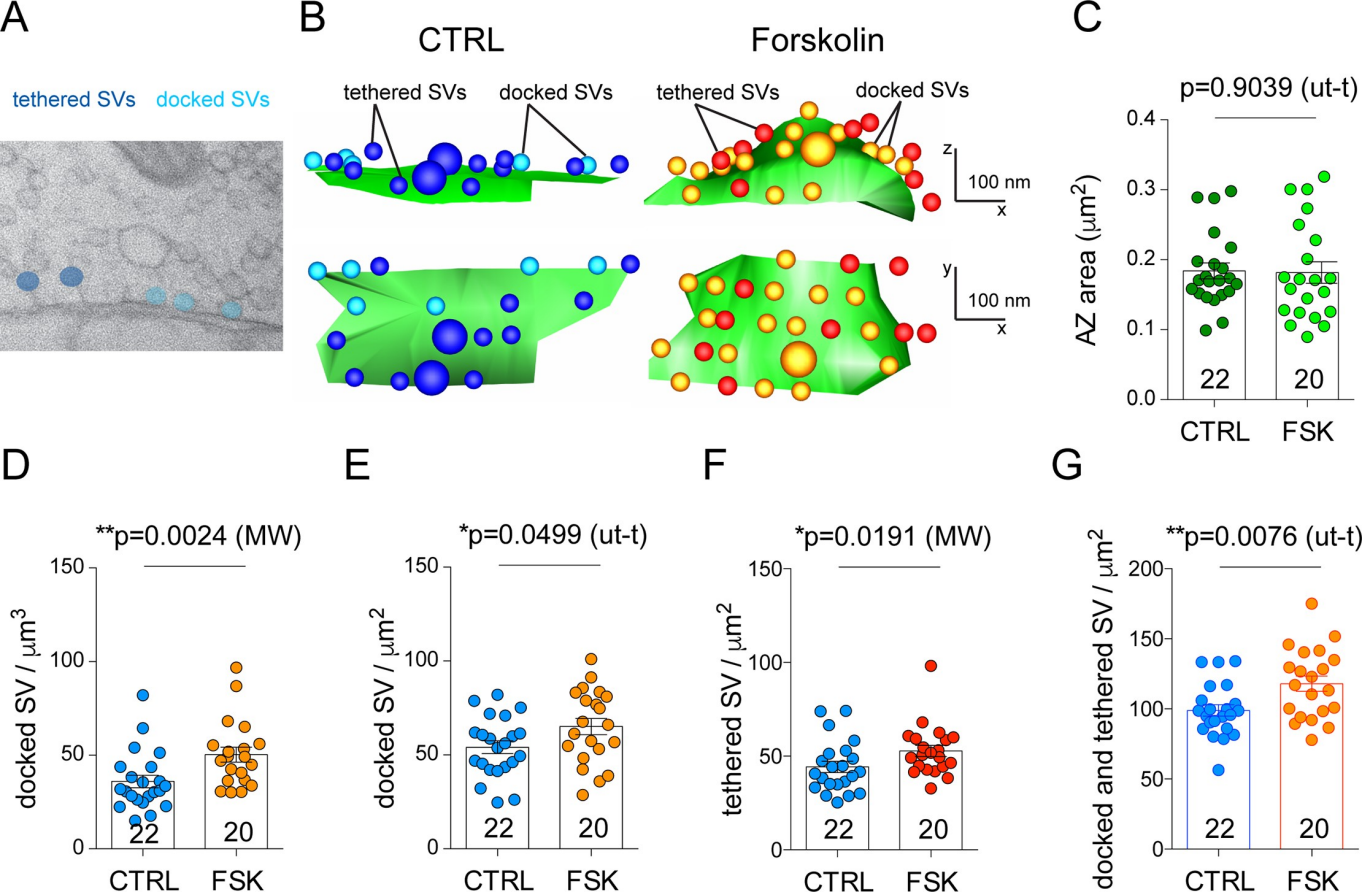
Docked vesicle density increases upon forskolin-induced potentiation. (A) Two-dimensional electron microscopy image from a high-pressure frozen mossy fiber AZ showing docked (light blue) and tethered (blue) SVs. (B) Three-dimensional reconstruction of mossy fiber AZs from acute slices cryo-fixed in control conditions or after forskolin treatment. Top panels show the *xz* views, and bottom panels the *xy* views. (C) Bar graph indicating the quantification of AZ area (μm^2^) for control and forskolin-treated boutons. (D–F) Bar graphs indicating the quantification of docked vesicle density in the whole bouton (docked SV/μm^3^) (D), docked vesicle density per square micron of AZ (docked SV/μm^2^) (E), and tethered vesicle density per square micron of AZ (tethered SV/μm^2^) (F) in control and forskolin-treated boutons. (G) Bar graph indicating the quantification of the putative readily releasable pool, measured as docked and tethered vesicle density per square micron of AZ (docked and tethered SV/μm^2^) in control and forskolin-treated boutons. Scatter points indicate the mean value for each individual bouton from 4 animals. Values represent mean ± SEM. AZ, active zone; CTRL, control; FSK, forskolin; MW, Mann–Whitney *U* test; SV, synaptic vesicle; ut-t, unpaired *t* test. The data underlying this figure can be found at doi: 10.5281/zenodo.4498214.

Vesicles physically docked at the AZ membrane are considered a good approximation of the readily releasable pool (RRP) of SVs [46]. Interestingly, physiological measurements of the RRP at hMFBs reported around 40 SVs per AZ [47], a value that is bigger than the morphologically docked pool and that can be approximated by the sum of vesicles whose center is found up to 60 nm from the plasma membrane [42] or by the sum of docked and tethered vesicles [48]. We asked whether, upon forskolin treatment, the increase in neurotransmitter release was paralleled by changes in the number of docked and tethered vesicles (Fig 6).

With HPF followed by EM imaging and 3D reconstruction of AZs, we observed an increase in docked vesicles per bouton (Fig 6D) as well as a 20% increase in docked vesicle density at individual AZs (Fig 6E). These values correspond to an average of 9.07 vesicles per AZ in control conditions and 10.25 vesicles per AZ after potentiation. To have an estimate of the morphological correlate of the RRP, we measured docked and tethered vesicles (Fig 6A and 6B). We observed a significant increase in the density of tethered vesicles and, consequently, in the sum of docked and tethered vesicles (putative RRP) in forskolin-treated samples compared to controls. Potentiated AZs on average displayed 118 ± 4.1 (mean ± SEM) vesicles per square micron, while controls had an average of 98.9 ± 5.5 (mean ± SEM) vesicles per square micron (Fig 6G). We also found a statistically significant increase in the number of docked vesicles per cubic micron of reconstructed boutons in chemically fixed slices, likely a consequence of the increase in the number of release sites visible in that preparation.

## Discussion

Our study elucidates the structural and functional modifications that underlie chemical presynaptic potentiation at hMFBs. Taken together, our data show that an increase in the number of available release sites—and not only in release probability—is instrumental for forskolin-induced mossy fiber presynaptic potentiation.

Growing evidence suggests that presynaptic plasticity may involve structural changes [28], and indeed, persistent increase in mossy fiber complexity has been shown to occur in mice kept in enriched environments [27].

Here we show that the recruitment of new release sites contributes to chemically induced presynaptic potentiation. This presynaptic unsilencing has been previously suggested by electrophysiological recordings of autaptic neurons [19] and by calcium imaging in cultured hippocampal slices [20]. Our EM and glutamate imaging analyses indicate that an increase in AZ and release site number leads to the increase in neurotransmission. EM of potentiated hMFBs revealed an increase in synaptic complexity, in AZ density (Fig 4), and in the morphological correlate of the RRP (Fig 6). Moreover, we gained evidence for an increase in the presynaptic releasing area by live 2-photon imaging of the glutamate sensor iGlu_u_. In our experimental conditions, the structural changes occurred already after 15 min of incubation in forskolin. This indicates that structural rearrangements occur in a short time frame and, if maintained, could consolidate long-term changes in synaptic strength. A similar time course of structural synaptic remodeling was observed at *Drosophila* neuromuscular junctions: There, rapid AZ remodeling, possibly implicating the insertion of AZ molecular scaffolds resulting in the incorporation of new release sites, has been shown to consolidate presynaptic potentiation and to sustain long-term changes in synaptic strength [49,50]. Our data thus suggest mechanistic conservation in the mechanisms of presynaptic long-term plasticity.

At cerebellar climbing fiber–Purkinje cell synapses, cAMP/PKA stimulation shifts the balance from univesicular to multivesicular release without affecting *P*_r_ [18]. By direct monitoring of glutamate release at hMFBs, we observed a forskolin-mediated decrease in the PPR of the released glutamate (Fig 2G), consistent with the previous notion of a forskolin-mediated increase in vesicular *P*_r_ [20,34]. Our experiments demonstrate that forskolin increases the active area without changing the amplitudes of the iGlu_u_ transients (Fig 2A–2D and 2I–2L). This is rather inconsistent with a switch from uni- to multivesicular release mode, which would imply an elevation of the peak glutamate concentration at the presynaptic membrane and/or in the synaptic cleft. However, we cannot exclude that a shift from uni- to multivesicular mode might occur at lower concentrations of extracellular calcium, as previously described for hMFBs [31]. Interestingly, we occasionally observed paired-pulse facilitation of maximal iGlu_u_ transients under control conditions at the single pixel level (Fig 1E–1H and 1K), while on the whole bouton level, the maximal amplitude invariably showed a paired-pulse depression (Fig 2G). This observation suggests that a switch from uni- to the multivesicular release per se might exist at these synapses that, however, is not prominently induced by forskolin at 2 mM extracellular Ca^2+^. Finally, potentiation was not associated with a decrease in glutamate clearance (Fig 2H) or an increase in the bouton size (Fig 1I). These data collectively suggest that forskolin mediates an increase in release site density. Nevertheless, the observed increase in AZ density (Fig 4D) cannot fully account for the 170% increase in the releasing area measured by glutamate imaging (Fig 2).

We could confirm recent findings [36] (Fig 1E–1H and 1K) that hMFBs display multiple sites of stochastic release. Such a feature might enable synapses to strongly facilitate release by switching from a random, low-probability mode of release to a more synchronous, high-probability mode at multiple AZs. Unfortunately, diffraction-limited 2-photon microscopy does not allow us to directly visualize release from single AZs. We attempted to unravel such synchronization by observing forskolin-induced changes in 2D patterns of iGlu_u_ transients. We found less entropy reduction (pixel randomness; Fig 2O) and less increase of non-triviality (pixel anisotropy; Fig 2P) in the presence of forskolin. It can be assumed that small (32% for entropy and 25% for non-triviality) but significant differences in these parameters were probably due to the 38%–46% addition of new AZs. At the release peak a high fraction of pixels in the image have a high intensity, and the addition of “bright” pixels may increase pixel homogeneity in the image. This might explain the small entropy decrease and the non-triviality increase that we observed (Fig 2M–2P). However, such an increase in pixel homogeneity may be due to equal changes induced in active pixels (pixel synchrony) due to glutamate release. Our measurements show no correlation between the active area and changes of entropy and non-triviality (S1G Fig), indicating that entropy and non-triviality are sensitive to global pixel synchrony rather than to local changes at single release sites. However, these results do not exclude the forskolin-mediated insertion of new AZs. Based on EM and glutamate imaging data, we propose that both processes (AZ insertion and the synchronization of multiple release sites, probably by increasing release probability) could be involved in hMFB LTP. The forskolin-induced simultaneous activation of multiple AZs can be interpreted as a forskolin-mediated decrease of the probabilistic pool of silent release sites or simply as an activation of silent release sites, as suggested before [19,20].

Synchronization of release sites requires an extended pool of vesicles ready to be released. Indeed, by EM, we observed an increase in the number of docked and tethered vesicles in forskolin-treated hMFBs. A similar PKA-dependent increase in docked vesicles has been recently observed at hMFBs after a high-frequency stimulation, and it has been proposed to constitute a “pool engram” that sustains post-tetanic potentiation and, possibly, short-term memory [17]. Further studies will be needed to determine whether the regulation of the RRP is responsible solely for short-term plasticity or whether it might also underlie the longer form of plasticity and memory.

Following adenylyl cyclase activation, we observed that SVs were more dispersed inside hMFBs. We do not know the molecular mechanism that regulates such dispersion. An educated guess would be that synapsin phosphorylation is mediating such dispersion, favoring the increase in the RRP size. In fact, vesicle clustering at the presynaptic terminal is known to be mediated by the synapsin family of proteins [51,52] and synapsins contain a conserved PKA phosphorylation site (Serine9) [53] that, when phosphorylated, mediates synapsin dissociation from vesicles [54]. PKA- and synapsin-mediated modulation of vesicle availability has also been observed in cultured human neurons [43].

We speculate that the dispersion of vesicles, their reorganization in the terminal, and the increase in the number of vesicles attached to the AZ are instrumental for the increase in release of neurotransmitter in the potentiated state.

Recent evidence implicates a direct role of nano-scale SV remodeling also as a presynaptic mechanism for Hebbian forms of plasticity [55]. It seems that the effect of forskolin on SV dispersion and mobilization mimics a more general mechanism that synapses adopt to modulate presynaptic performance, and forskolin effects might differ at different synapses depending on the variety of presynaptic molecular architecture of release sites.

The shortening of the coupling distance between presynaptic calcium channels and release sites has also been proposed to mediate the increase in neurotransmitter release at potentiated hMFBs [29]. We tested this hypothesis and performed gSTED microscopy to measure the distance between Cav2.1 and Munc13-1 signals. We confirmed a rather loose coupling distance of about 65 nm between calcium source and release sites at mossy fibers, as previously estimated by electrophysiological recordings [27] and gSTED microscopy [40]. These values were similar for control and potentiated synapses, suggesting that the tightening of the distance between calcium source and release sites does not underlie presynaptic potentiation. We are aware that the change in distance that we seek to measure is close to the resolution limit for our technique. However, we could measure a shorter coupling distance, of about 55 nm, between Cav2.1 and Munc13-1 in the area CA1, indicating that with our experimental design we can, in principle, resolve shorter distances (S4 Fig). We did not analyze the distance between other calcium channel types (N-type, R-type) and Munc-13. Further experiments will be needed to determine whether their reorganization might contribute to hMFB forskolin-mediated potentiation.

In summary, our results demonstrate that elevating cAMP at hMFBs increases their morphological complexity, recruits new AZs, and prepares the release machinery for synchronous release from multiple release sites, seemingly without altering the distance between calcium channels and release sites. The rapid structural remodeling and the increased release synchrony may thereby support the presynaptic expression of LTP at mossy fiber synapses.

Activity-dependent ultrastructural changes have been recently investigated at hMFBs by flash-and-freeze EM [56,57]. This technique, combined with the new optogenetic tool synaptoPAC that drives light-induced adenylyl cyclase activation [58], will help to unravel the differential effect of chemically induced and action-potential-evoked presynaptic potentiation at hMFBs.

## Methods

### Ethics statement

All animal experiments were carried out according to the guidelines stated in Directive 2010/63/EU of the European Parliament on the protection of animals used for scientific purposes and were approved by the animal welfare committee of Charité–Universitätsmedizin Berlin and the Landesamt für Gesundheit und Soziales–Berlin (permit T 0100/03).

### Chemical potentiation induction

Chemical presynaptic potentiation was induced in organotypic and acute slices by incubating slices from the same animal at room temperature for 15 min in either 50 μM forskolin in artificial cerebrospinal fluid (ACSF) or in ACSF + DMSO (1:1,000) as a control.

### Organotypic cultures of mouse hippocampus

Organotypic hippocampal slice cultures were prepared as described previously [59]. Briefly, postnatal day 3–8 C57BL/6N male mice were anesthetized by isoflurane, and the brain was removed and placed in ice-cold sterile slicing solution consisting of 50 mM sucrose, 87 mM NaCl, 2.5 mM KCl, 1.25 mM NaH_2_PO_4_, 26 mM NaHCO_3_, 3 mM MgCl_2_, 0.5 mM CaCl_2_ and 10 mM glucose. Horizontal brain slices (350 μm) were prepared with a vibratome (VT1200 V, Leica Microsystems) and placed on 30-mm hydrophilic PTFE membranes with 0.4-μm pores (Merck, Millipore, Ireland). Membranes were inserted into 35-mm petri dishes containing 1 mL of culture medium, and cultures were maintained up to 25 d in an incubator at 37°C, 95% O_2_/5% CO_2_. Culture medium was replaced 3 times per week and contained 50 mL of Basal Medium Eagle, 25 mL of Hanks’ balanced salt solution, 25 mL of horse serum, 0.5 mL of GlutaMAX-I Supplement (200 mM), and 2.5 mL of glucose (6 g/l). One day after preparation, the medium was supplemented with 0.5 mL of penicillin/streptomycin.

### Autaptic cultures

Autaptic cultures were prepared as previously described [60]. Briefly, hippocampi from postnatal day 1 C57BL/6N mice were removed and placed in cold Hanks’ balanced salt solution. The dentate gyrus was separated from the hippocampus and digested with Papain solution. Subsequently, cells were manually titrated and plated on glia cell islands grown on 30-mm coverslips in 6-well plates with a density of 4.95 × 10^2^ cells/cm^2^. Wells contained 3 mL of Neurobasal-A (Thermo Fisher Scientific, #10888022) supplemented with 2% B27 (Thermo Fisher Scientific, #17504001) and 0.2% penicillin/streptomycin (Thermo Fisher Scientific, #15140122).

### Viral transduction

#### Organotypic cultures

One day after the preparation, slice cultures were transduced with adeno-associated virus (AAV) serotype 9 particles encoding CaMKII.iGlu_u_.WPRE-hGH [30]. AAV particles were produced by the Viral Core Facility of the Charité–Universitätsmedizin Berlin (5.88 × 10^12^ genome copies/mL). Two hundred nanoliters of the virus suspension was injected into the hippocampal dentate gyrus under sterile conditions through a 20-μm glass capillary fixed on a mechanical manipulator under visual control through a binocular. The capillary was connected to a 5-μl Hamilton syringe. After transduction, cultures were incubated for at least 2 wk before being used for experiments. Because iGlu_u_ stains the plasma membrane, the somata of hippocampal granule cells appear dark in contrast to the bright dendritic tree and axons (Fig 1A and 1D). Despite the dense packing of hMFBs in the stratum lucidum of the CA3 area, this type of transduction, in combination with 2-photon imaging, does not allow the visualization of more than 1 bouton in a 20-μm^2^ view field (Fig 1D).

#### Autaptic cultures

One to 2 d after the preparation, autaptic cultures were transduced with 2 μl of the AAV encoding CaMKII.iGlu_u_.WPRE-hGH per well.

### Quantification of elevation of synaptic glutamate concentration with iGlu_u_

Glutamate release from single hMFBs was visualized using the genetically encoded ultrafast glutamate sensor iGlu_u_ [30], which has been used for high-speed glutamate imaging before [32,33,61]. The main advantages of this sensor are its low sensitivity (Kd = 600 μM) and fast decay kinetics, which allow only intrasynaptic glutamate with good temporal resolution to be visualized and eliminate the registration of glutamate signals from neighboring synapses. To image synaptically released glutamate, transduced organotypic hippocampal cultures were submerged into a perfusion chamber with a constant flow of oxygenated ACSF at a rate of 1–2 mL/min. ACSF contained 120 mM NaCl, 2.5 mM KCl, 1.25 mM NaH_2_PO_4_, 25 mM NaHCO_3_, 10 mM glucose, 2 mM CaCl_2_, and 1 mM MgCl_2_, with pH 7.3 and osmolarity 300 mOsm/L. Temperature during the recordings was maintained at 32–35°C.

A Femto2D 2-photon laser scanning system (Femtonics, Budapest, Hungary) equipped with a femtosecond pulsed Ti:Sapphire laser tuned to λ = 805 nm and power 0.5 W (Cameleon, Coherent, Santa Clara, CA, US) controlled by the MATLAB-based MES software package (Femtonics, Budapest, Hungary) was used for the excitation of iGlu_u_ expressed at hippocampal mossy fibers (Fig 1A and 1D). Fluorescence was acquired in epifluorescence mode with a water immersion objective (LUMPLFL 60×/1.0 NA or UMPlanFL 10×/0.3 NA, Olympus, Hamburg, Germany). Transfluorescence and transmitted infrared were detected using an oil immersion condenser (Olympus).

At rest, the low-affinity iGlu_u_ sensor produces a weak fluorescence (480–600 nm) indistinguishable from autofluorescence (Fig 1B). To discriminate between iGlu_u_-positive structures and autofluorescent elements (Fig 1C) that emit light in the whole visible spectral range, fluorescent photons from both green (<600 nm) and red (≥600 nm) spectral bands were collected simultaneously, but separately with 2 photomultipliers (Fig 1B–1D). hMFBs were identified by the following criteria (Fig 1B–1D): (1) fluorescence in the green but not in the red spectral range, (2) a round form with an approximate diameter < 6 μm, (3) the form being connected to a clearly visible axon, and (4) green fluorescence increase in response to electrical stimulation.

In order to evoke glutamate release from hMFBs, we electrically stimulated an axon connected to the boutons with pairs of a negative rectangular current pulse (≤5 μA, generated with the Isolator-11, Axon Instruments, US) through a unipolar glass electrode filled with ACSF (tip diameter 1 μm, resistance 8 MΩ). The interstimulus interval in pairs was 50 ms. The stimulation electrode was placed on the axon in vicinity (<20 μm) of the bouton (Fig 1D). For measurements of the virtual bouton diameter (diameter of a circle with area equal to the area of the recorded bouton), we used images of big view fields (100 × 100 μm^2^) with a spatial resolution of 0.1 μm/pixel, which we acquired by averaging 15 individual frames at the confocal plane where the bouton had the largest iGlu_u_-positive area.

The iGlu_u_ fluorescence signal was acquired at a frequency of 1.6 kHz from a rectangular region of interest (ROI) covering the whole bouton at the confocal plane with maximal bouton area. The scanning pattern and mean spatiotemporal scanning characteristics are shown in Fig 1J. These characteristics varied for each individual recording, with a coefficient of variation of 35% to reach maximal resolution for each bouton, but they were not significantly different under different conditions. The analysis of fluorescent signal was performed with a custom-made routine. To evaluate evoked responses, signals for all pixels of the ROI were filtered with a 100-Hz low-pass filter and evaluated separately. The iGlu_u_ pixel signal was expressed as a change of fluorescence intensity (Δ*F*) in percent of the mean baseline fluorescence *F* (Δ*F/F*) for the given pixel. The baseline was determined as the data points acquired during a 50-ms period prior to stimulation (baseline). For the construction of time- and space-dependent Glu concentration profiles after evoked release, suprathreshold pixels were determined, the threshold being defined as 3 × SD of Δ*F/F* (Figs 1F–1H, 1K, 2A and 2B). The stimulus-induced changes of suprathreshold Δ*F/F* in time or space are referred to as “iGlu_u_ transients.”

To assess the dynamic characteristics of the iGlu_u_ signal, the area occupied by suprathreshold pixels (active area) (Fig 2A and 2B) and the pixel intensities expressed as Δ*F/F* were plotted against time (Fig 2C, 2E, 2I and 2K). Peak values (Fig 2D, 2F, 2J and 2L) and their PPRs (Figs 2G, S1B and S1C) were determined for the active area, the cumulative amplitude (spatial integral of intensities for suprathreshold pixels), the maximal amplitude (maximal intensity for a population of suprathreshold pixels), and the mean amplitude (mean intensity for a population of suprathreshold pixels) of the iGlu_u_ transients. The mean amplitude indicates the mean concentration of glutamate in the synaptic cleft, and the maximal amplitude refers to the glutamate concentration near release sites. The tau of decay (τ) is the time constant of decay derived by fitting a monoexponential function to the decay from the peak of the cumulative iGlu_u_ transients (Fig 2F). Data analysis was performed blinded to the experimental condition.

### Entropy and non-triviality measurements

The main idea of the non-triviality–entropy analysis is to quantify the spatial properties of representative 2D Δ*F/F* images with respect to their balance between randomness and structural order, triviality, and non-triviality. Highly ordered structures (like a grid) have near-zero entropy and near-zero non-triviality. In contrast, completely disordered structures (e.g., independent and identically distributed Gaussian noise) have maximal entropy and very small non-triviality. Intermediate values of entropy are associated with higher values of non-triviality if the underlying pattern contains features with preferred orientation [62,63]. In our analysis, informational entropy characterizes the homogeneity of 2D patterns, and non-triviality at high entropy characterizes their anisotropy. A detailed theoretical overview of the analysis is described in a method paper [37], and an implementation Python code is available at doi: 10.5281/zenodo.1217636. To avoid overlapping terms in this paper, we have replaced the originally published term “complexity” with its synonym “non-triviality.”

### Electrophysiological recordings in autaptic cultures

To control for the effect of the expression of iGlu_u_ on neurotransmission, we performed electrophysiological recordings of autaptic hippocampal neurons transduced with an AAV encoding for iGlu_u_. Neurons were recorded at day in vitro 14–17 at room temperature on an IX73 inverted microscope (Olympus, Shinjuku, Tokyo, Japan) using a Multiclamp 700B amplifier under the control of a Digidata 1550 AD board and Clampex 10 software (all Molecular Devices). Data were acquired at 10 kHz and filtered at 3 kHz, and series resistance was compensated at 70%. The extracellular solution contained 140 mM NaCl, 2.4 mM KCl, 10 mM HEPES, 10 mM glucose, 2 mM CaCl_2_, and 4 mM MgCl_2_ (pH adjusted to 7.3 with NaOH, 300 mOsm/L). The intracellular solution contained 136 mM KCl, 17.8 mM HEPES, 1 mM EGTA, 4.6 mM MgCl2, 4 mM Na2ATP, 0.3 mM NaGTP, 12 mM disodium phosphocreatine, and 50 U/mL creatine phosphokinase (pH adjusted to 7.3 with KOH, 300 mOsm/L). Chemicals were purchased from Tocris, Merck, or Carl Roth. Autaptic neurons were recorded in whole-cell voltage clamp mode using thick-walled borosilicate pipettes (3–4 MΩ). Membrane potential was set to −70 mV. Paired EPSCs were evoked every 5 s by triggering 2 unclamped action potentials with 40-ms interstimulus interval using 1-ms depolarizations of the soma to 0 mV. The PPR was calculated as the ratio of the second to the first EPSC amplitude. Data were analyzed using AxoGraph X (AxoGraph, Sydney, Australia). EPSC slope was determined by a linear fit to the average EPSC signal of 6 sweeps, and the EPSC decay time constant was determined by a monoexponential fit. We could not observe any difference in synaptic transmission between iGlu_u_-expressing neurons and non-transduced cells (S2 Fig).

### Immunocytochemical staining of autaptic cultures

Transduced and non-transduced cultured hippocampal neurons were fixed at room temperature in 4% paraformaldehyde (PFA) for 15 min at day in vitro 21. Afterwards, cultures were washed in 0.1 M phosphate buffer saline (PBS), permeabilized with PBS containing 0.1% Tween20 (PBS-T) and quenched with PBS containing 100 mM glycine. Cells were blocked for 30 min in PBS containing 5% normal goat serum and then incubated with primary antibodies at room temperature for 1 h (chicken anti-GFP [1:1,000] and rabbit anti-MAP2 [1:1,000]) (Table 1). After thorough washing with PBS-T, secondary antibodies were incubated at room temperature for 1 h in the dark (goat anti-chicken Alexa Fluor 488 [1:1,000] and goat anti-rabbit Alexa Fluor 594 [1:500]). After several washing steps with PBS-T and PBS, cultures were mounted on coverslides in Mowiol (Carl Roth, #0713.1) and cured in the dark for 18 h.

**Table 1:** Antibodies used in immunocytochemical staining.

Target molecule	Primary antibody	Secondary antibody
GFP	Chicken-α-GFP, (Abcam, #13970), 1:1,000	Goat-α-chicken–Alexa488 (Invitrogen, #11039), 1:1,000
MAP2	Rabbit-α-MAP2 (Merck Millipore, #AB5622), 1:1,000	Goat-α-rabbit–Alexa594 (Invitrogen, #A-11012), 1:500

### Confocal microscopy

Cured mounted cultures were imaged using a confocal microscope (Leica TCS SP5), equipped with a 40× oil immersion objective. Images were acquired with the LAS AF software (Leica Microsystems). For excitation of Alexa Fluor 488, counterstaining the GFP molecule from the iGlu_u_SNFR construct, an argon laser at 488 nm was used with 10% power. For excitation of Alexa Fluor 594, a DPSS laser at 561 nm was used with 10% power. Emission was detected by 2 photomultiplier tubes. Laser power and gain were kept the same for all imaged cultures and conditions. *Z*-stacks were acquired in a sequential line-by-line mode in *z*-steps of approximately 1 μm, with a speed of 200 Hz and a line average of 4.

Immunofluorescence images depict the summed fluorescence signal from the *z*-stacks. Brightness, intensity, and contrast were adjusted, and colors were inverted.

### Acute slice preparation

Postnatal day 27–29 male WT C57BL/6N mice were anesthetized with isoflurane and decapitated, and brains were quickly removed and placed in ice-cold sucrose–ASCF (s-ACSF) containing 50 mM NaCl, 25 mM NaHCO_3_, 10 mM glucose, 150 mM sucrose, 2.5 mM KCl, 1 mM NaH_2_PO_4_, 0.5 mM CaCl_2_, and 7 mM MgCl_2_. All solutions were saturated with 95% O_2_/5% CO_2_ (vol/vol) (pH 7.4).

For STED microscopy, hemispheres were embedded in 4% low-melt agarose in HEPES-buffered solution. Sagittal slices (100 μm for STED microscopy, 350 μm thick for conventional EM, and 150 μm thick for HPF) were cut with a vibratome (VT1200 V, Leica Microsystems) in ice cold s-ACSF solution, stored submerged in s-ACSF for 30 min at 35°C (at room temperature for STED microscopy), and subsequently stored at room temperature in ACSF containing 119 mM NaCl, 26 mM NaHCO_3_, 10 mM glucose, 2.5 mM KCl, 1 mM NaH_2_PO_4_, 2.5 mM CaCl_2_, and 1.3 mM MgCl_2_ saturated with 95% O_2_/5% CO_2_ (vol/vol) (pH 7.4). Experiments were started 1 to 3 h after the preparation. For STED microscopy, slices were fixed with 4% PFA in PBS for 1 h at room temperature immediately after the induction of chemical potentiation and were later stored in PBS + 0.1% NaN_3_ for up to 4 d until staining.

### Immunohistological staining for STED microscopy

After PFA fixation, slices were washed in 0.1 M phosphate buffer (PB) containing 20 mM glycine. They were incubated for 3 h in a blocking solution containing 10% normal goat serum and 0.3% TritonX-100 in PB. After rinsing with 0.3% TritonX-100 in PB, a second blocking step was performed with goat Fab fragments anti-mouse IgG (1:25) in PB for 1 h at room temperature. After rinsing with 0.3% TritonX-100, primary antibodies (mouse anti-ZnT3 [1:500], chicken anti-Homer1 [1:200], guinea pig anti-Cav2.1 [1:500], and rabbit anti-Munc13-1 [1:150]) (Table 2) were incubated on a shaker at 4°C for 40 h in PB containing 5% normal goat serum and 0.3% TritonX-100.

**Table 2:** Antibodies used in immunohistological staining.

**Target molecule**	**Primary antibody**	**Secondary antibody**
ZnT3	Mouse-α-ZnT3, Synaptic Systems, #197 011), 1:500	Goat-α-mouse–Alexa405 (Invitrogen, #31553), 1:200
Homer1	Chicken-α-Homer1 (Synaptic Systems, #160 006), 1:200	Goat-α-chicken–Alexa488 (Invitrogen, #11039), 1:200
Cav2.1	Guinea pig-α-Cav2.1 (Synaptic Systems, #152 205), 1:500	Goat-α-guinea pig–Alexa594 (Invitrogen, #A-11076), 1:100
Munc13-1	Rabbit-α-Munc13-1 (Synaptic Systems, #126 102), 1:150	Goat-α-rabbit–ATTO647N (Activ Motif, #15048), 1:200

Over a period of 3 h at room temperature, slices were washed in 0.3% TritonX-100 in PB every 15–20 min. Secondary antibodies (goat anti-rabbit ATTO647N [1:200], goat anti-mouse Alexa Fluor 405 [1:200], goat anti–guinea pig Alexa Fluor 594 [1:100], and goat anti-chicken Alexa Fluor 488 [1:200]) were centrifuged at 4°C and 300 relative centrifugal force for 30 min. Then, slices were incubated with secondary antibodies in PB containing 5% normal goat serum and 0.3% TritonX-100 for 2 h on the shaker, in the dark and at room temperature.

After washing, slices were mounted on superfrost coverslides (VWR), embedded with Prolong Gold (Thermo Fisher Scientific), covered with high-precision coverslips (Carl Roth), and cured for 24 h at room temperature in the dark. STED imaging was performed after 5–7 d to ensure the best refractive index for Prolong Gold. Imaging in CA1 for a subset of the data (5 out of 11 animals) was performed more than 40 d after the staining.

### STED microscopy imaging

Cured slices were checked for ZnT3-Alexa405 staining, which specifically labels the mossy fiber band, using a confocal microscope (Leica TCS SP5). Slices were imaged with a time-gated STED (gSTED) setup (Expert Line, Abberior Instruments, Germany) equipped with an inverted IX83 microscope (Olympus) and a 100×, 1.40 NA oil immersion objective. Images were acquired using the Imspector software (version 16.1.6477, Abberior Instruments, Germany).

After orientation in the slice, imaging areas in CA3 or CA1 were chosen. Overview images of 75 × 75 μm were scanned in confocal mode. Within this overview, several ROIs of 10 × 10 μm were chosen for scanning in STED mode. In CA3, scanning was performed in stratum lucidum, close to CA3 pyramid cell bodies (see S4E Fig). In CA1, scanning was performed in stratum radiatum more distal from the pyramidal cell bodies (see S4F Fig). Sixteen-bit 2D gSTED images were acquired within chosen areas with a pixel size of 20 × 20 nm, a laser dwell time of 2 μs, and a line accumulation of 10 (confocal mode) or 30 (gSTED mode). Pulsed excitation lasers had wavelengths of 640 nm, 561 nm, and 488 nm. The dyes ATTO647N and Alexa594 were depleted first, using a pulsed gSTED laser at 775 nm (0.98-ns pulse duration, up to 80-MHz repetition rate). Subsequently, Alexa Fluor 488 was depleted using a pulsed gSTED laser at 595 nm (0.52-ns pulse duration, 40-MHz repetition rate). Time gating was set to 750 ps. Avalanche photodiode detectors collected fluorescence signals sequentially in a line-by-line mode. In parallel to gSTED scanning, confocal images were acquired. One slice per condition and mouse was imaged with the gSTED microscope. Per slice, 6–8 images were scanned. After 595-nm STED imaging, we verified the localization of the bleached ROIs in the ATTO647N channel within ZnT3-positive regions using a confocal microscope (Leica SP5; see S4E and S4F Fig). With our approach, we cannot exclude that a small fraction of the distances measured do not belong to hMFBs. Nevertheless, this eventuality remains unlikely considering that, in the CA3 stratum lucidum, most excitatory/asymmetric synapses are formed by mossy fibers onto CA3 thorny excrescences [64]. In our study, we used Homer1 as marker for excitatory synapses, to further restrict our analysis to hMFBs.

### STED microscopy analysis

Raw triple-channel gSTED images were deconvolved for quantification with the Imspector software (version 16.1.6477, Abberior Instruments, Germany) using the Richardson–Lucy algorithm. The point spread function had a full width at half maximum of 40 nm, based on measurements with 40-nm Crimson beads, and was computed with a 2D Lorentzian function.

#### Coupling distance measurement

Deconvolved 32-bit gSTED images were merged with Fiji (ImageJ version 1.52n) to a triple-channel composite. Up to 17 synapse configurations (i.e., triads of juxtaposed Cav2.1, Munc13-1, and Homer1 signals) were manually chosen in each composite. The intensity maxima of Cav2.1 and Munc13-1 were retrieved using the Fiji “Find maxima” tool with a prominence > 20, except for a subset of CA1 data where the prominence was > 10. The intensity maxima were output as point selections and used to measure the distance between Cav2.1 and Munc13-1 clusters: With the Fiji tool for straight lines (size: 1 pixel), a line was drawn through both intensity maxima. Then the “modified multichannel plot profile” plugin (written by Tiago Ferreira) was used to plot the intensities of all 3 channels along this line, meaning that each intensity value corresponded to a distance value on the line. Based on this, the distance was calculated between the locations of the intensity maxima of the Cav2.1 and Munc13-1 signals. Data acquisition and analysis were performed blinded to the experimental condition.

### Conventional EM

After the induction of chemical potentiation, 350-μm-thick acute slices were immersed in a solution containing 1.2% glutaraldehyde in 66 mM sodium cacodylate buffer for 1 h at room temperature.

After washes in 0.1 M sodium cacodylate buffer, slices were then postfixed in 2% OsO_4_ in dH_2_O for 1 h at room temperature. Slices were then washed and en bloc stained with 1% uranyl acetate in dH_2_O and dehydrated in solutions with increasing ethanol concentration. Final dehydration was obtained incubating slices in propylene oxide, and then the infiltration of Epoxy resin was obtained by serial incubations in increasing resin/propylene oxide concentrations. Samples were finally flat embedded in epoxy resin (Epon 812 Kit, Science Services) for 48 h at 60°C.

Serial sections of 70 nm were cut with an UltraCut ultra microtome (Leica) equipped with a 45° Ultra Diamond Knife (Diatom) and collected on formvar-coated copper slot grids (Science Services).

### HPF and freeze substitution

After the induction of chemical potentiation, 150-μm-thick acute slices of hippocampi were dissected and placed in a 3-mm aluminum HPF Carrier Type A (Science Services). Samples were cryo-fixed using a HPF machine (EM ICE, Leica) in ACSF (with or without forskolin). A drop of 20% BSA in ACSF was added for cryo-protection and a 3-mm aluminum HPF Carrier Type B (Science Services) was placed on top of the sample prior to HPF. Frozen samples were transferred in a freeze substitution machine (AFS2, Leica) and placed in a solution containing 2% OsO_4_ and 0.4% uranyl acetate in anhydrous acetone at −90°C. The following substitution protocol was performed: Samples were kept at −90°C for 54 h, then temperature was brought from −90°C to −60°C in 6 h, held at −60°C for 8 h, and then raised to −30°C in 6 h. Subsequently, temperature was held for 8 h at −30°C and then brought to 0°C in 4 h. At 0°C, samples were washed in anhydrous acetone and slowly infiltrated in increasing concentrations of Epon in acetone. The last infiltration steps were carried out at room temperature in pure Epon and were followed by embedding at 60°C for 48 h.

HPF of acute slices is challenging because an acute slice’s minimal slice thickness is similar to the maximal thickness compatible with HPF (200 μm), and this often results in suboptimal sample freezing and/or vibratome damage [57]. Despite this drawback, acute slice preparation is a good way to preserve the tissue in conditions that are crucial for the read-out of physiological phenomena such as presynaptic plasticity [17,56].

### EM imaging of serial sections and 3D reconstructions

The CA3 region of the hippocampi was identified by semi-thin sectioning and toluidine blue staining for light microscopy observation. When the CA3 region was clearly visible, the ROI was trimmed, and 70-nm ultrathin serial sections were collected on formvar-coated copper slot grids (Science Services). Imaging was performed with an EM 900 Transmission Electron Microscope (Zeiss) operating at 80 kV and equipped with a 2K digital camera (Olympus). Initially 2D imaging was performed with a Tecnai G20 (Thermo Fisher Scientific) operated at higher voltage (120–200 kV), but for our serial sections, we opted for 80-kV imaging in order to have a better contrast. We focused the imaging on the stratum lucidum of the hippocampal region of the CA3, because the presence of big mossy fiber boutons was easily distinguishable and because the stratum lucidum is located just above the pyramidal cell layer. Serial images of the same mossy fiber boutons were manually acquired using ImageSP software and were aligned using the midas script of IMOD software, and for each bouton, synaptic profiles and all organelles were manually segmented in each image. Data acquisition and analysis were performed blinded to the experimental condition.

### Statistics

Data are shown as mean ± SEM. For statistical analysis, all datasets were tested for normality using the D’Agostino and Pearson normality test. For comparisons between normally distributed datasets, we performed a 2-tailed unpaired *t* test. If the variance was significantly different between the compared datasets, *t* tests were performed with Welch correction. For non-normally distributed data, we performed a 2-tailed non-parametric Mann–Whitney *U* test comparing the ranks of treated synapses and controls. Cumulative distributions were tested using the 2-sample Kolmogorov–Smirnov test. We used Prism 6.2 and 8.4 software (GraphPad) for the analysis. Levels of significance are indicated in the figures as *p < 0.05, **p < 0.01, ***p < 0.001, and ****p < 0.0001.

## Supporting information

S1 Fig Correlograms for different parameters of iGlu_u_ transients acquired from hMFBs under control conditions. (A–C) Correlograms of cumulative amplitude (A), mean amplitude (B), and active area (C) versus their paired-pulse ratios demonstrate that among others cumulative amplitude (A) best reflects the activity-dependent form of short-term plasticity. (D–F) Correlograms of active area versus cumulative (D), mean (E), and maximal (F) amplitudes show that the active area is independent of glutamate concentration within the synaptic cleft (E and F), but is associated with the total amount of released glutamate (D), i.e., the measured active area reflects more a releasing area than a diffusional glutamate spread. (G–I) Correlograms of active area (G), mean amplitude (H), and cumulative amplitude (I) versus entropy and non-triviality change provide evidence that entropy and non-triviality are independent of the active area and amount of released glutamate. The data underlying this figure can be found at doi: 10.5281/zenodo.4498214.
(TIF)

S2 Fig Analysis of synaptic transmission in autaptic cultures of hippocampal neurons revealed no difference between neurons expressing iGlu_u_ and noninfected control neurons. (A) Immunofluorescence images of cultured hippocampal neurons PFA-fixed at day in vitro 21 and stained for the dendritic marker MAP-2 and iGlu_u_. The iGlu_u_ signal was enhanced by immunolabeling with anti-GFP antibodies. (B) Exemplary traces of whole-cell voltage clamp recordings of excitatory postsynaptic currents (EPSCs) evoked by 1-ms depolarizations in autaptic cultures of a noninfected control neuron, or neuron transduced with an AAV encoding iGlu_u_. Fits for calculating the EPSC slope (red) and EPSC time constant (green) are shown as overlay. (C–E) EPSC amplitude (C), slope (D), and decay time constant (E) were not different between control and iGlu_u_-expressing neurons. (F) Example traces of 2 EPSCs evoked at 25 Hz in a control and an iGlu_u_-expressing neuron. (G) Typical synaptic currents evoked by brief applications of hypertonic sucrose solutions (500 mM) to deplete the readily releasable pool (RRP). (H–J) Paired-pulse ratio (PPR) (H), size of the RRP (I), and vesicular release probability (*P*_r_) (J) were not significantly different between control and iGlu_u_-expressing neurons. (K) Control neurons (open circles) and iGlu_u_-expressing neurons (black circles) showed similar short-term kinetics of release during 10-Hz stimulation. Control: *n* = 12–13; iGlu_u_: *n* = 12–14; *N* = 2 cultures for both conditions. The data underlying this figure can be found at doi: 10.5281/zenodo.4498214.
(TIF)

S3 Fig Longitudinal iGlu_u_ imaging experiments reveal forskolin effect on hMFB glutamate release. (A) Image sequence showing iGlu_u_ fluorescence for the hMFB recorded at different time points before (−12, −6, and 0 min) and after (6, 12, and 18 min) forskolin application. High-resolution images were taken before high-speed imaging. The green rectangle on the pictures marks the area of high-speed imaging. Note strong iGlu_u_ bleaching during recordings. (B) Sequence of pictures demonstrating Δ*F/F* spatial distributions at peak response to the first electrical stimulation acquired with high-speed imaging of marked (A) area for hMFB at different time points before (−12, −6, and 0 min) and after (6, 12, and 18 min) forskolin application. The contoured pixels represent the active area (pixels with response amplitude > 3 × SD of Δ*F/F* at rest). Note increased number of active pixels after forskolin application. (C) Graph demonstrating mean iGlu_u_ fluorescence signal *F* (black line and symbols) and mean standard deviation of Δ*F/F* at rest (gray line and symbols) for all pixels (*n =* 169) used for high-speed imaging. The data were calculated by averaging all values (mean F or SD of ΔF/F) calculated for each point of the baseline (50 ms prior to electrical stimulation). Data are normalized to the first value, 12 min before the forskolin application. Note 50% bleaching and 25% increased pixel noise. (D) Graph shows the cumulative amplitude, active area, and mean and maximal amplitudes of the iGlu_u_ fluorescent signal of the hMFB in (A and B). Data are normalized to the mean values of given parameters under control conditions. Note the forskolin-induced 200% increase in active area and cumulative amplitude, while mean and maximal amplitudes remain unaltered. (E) Graph demonstrating statistics for the active area (black line and symbols) and mean amplitudes (gray line and symbols) for 5 different hMFBs. Numbers in brackets show the number of data points used for averaging. Asterisks mark time points where the active area was significantly bigger than the active area at time point 0 min (significance was tested with paired *t* test, *p* < 0.05). Note the significant increase of the active area and the non-significant elevation of mean amplitudes. The latter tendency can be explained by the fact that the sensor bleaching leads to increased pixel noise, elevated threshold for active pixels, and decreased portion of low-amplitude pixels in the active pixel pool taken for averaging. The data underlying this figure can be found at doi: 10.5281/zenodo.4498214.
(TIF)

S4 Fig Coupling distance between Cav2.1 and Munc13-1 in CA1 is shifted towards smaller values. (A) Example scan in area CA1: confocal scan (top), raw gSTED scan (middle), and deconvolved gSTED scan (bottom). Staining for Cav2.1 (green), Munc13-1 (magenta), and Homer1 (cyan). (B) Example of an analyzed synapse in CA1: The distance between Cav2.1 (green) and Munc13-1 (magenta) was measured only if they were close to a Homer1-positive spot (cyan); line profiles were measured at the dotted line (top), drawn through the intensity maxima of the Cav2.1 and Munc13-1 signals (arrowheads). The distance was calculated between the intensity maxima of the Cav2.1 and Munc13-1 signals, shown in the corresponding normalized intensity plot (bottom). (C) Frequency distribution with a bin size of 20 nm for CA3 control (blue) and CA1 (yellow). The distribution of measured distances between Cav2.1 and Munc13-1 is different for CA1 compared to CA3 control. (D) Cumulative frequency with a bin size of 20 nm for CA3 control (blue) and CA1 (yellow). The cumulative distribution was significantly shifted to smaller values for CA1 compared to CA3 (*p* < 0.001, Kolmogorov–Smirnov test). (E) Imaging areas in CA3 were situated in the ZnT3-positive area. After STED imaging, images were taken at a confocal microscope to visualize the ZnT3 staining (i) and the imaging areas, which were bleached in the red channel by the second STED laser (ii) and situated within the ZnT3-positive mossy fiber band (iii) in the stratum lucidum, close to the CA3 pyramidal somata (iv). Imaging areas are indicated by black arrowheads. (F) Imaging areas in CA1. Confocal images were acquired after STED imaging to visualize the absence of ZnT3 in CA1 (i) as well as the bleached imaging areas in the red channel (ii), which were situated in area CA1 (iii) in stratum radiatum, with some distance to CA1 pyramidal somata (iv). Imaging areas are indicated by black arrowheads. The data underlying this figure can be found at doi: 10.5281/zenodo.4498214.
(TIF)

S5 Fig Three-dimensional EM analysis reveals an increase in presynaptic complexity and active zone density in forskolin-treated chemically fixed acute slices. (A) Electron microscopy image of the stratum lucidum of the hippocampal CA3 region. A pyramidal cell soma (pyr) and mossy fiber axon bundles (mf) are visible in the left panel. In the central panel large presynaptic terminals contacting multiple spine heads (sp) are visible. The right panel shows a high-magnification image of 3 AZs. (B) Partial 3D reconstruction computed from manually segmented serial images of hMFBs in control conditions (CTRL) or after forskolin treatment. Presynaptic membrane is green, postsynaptic membrane is light blue, synaptic vesicles are yellow, and active zones and docked vesicles are blue (control) or red (forskolin). (C) Bar graph indicating the quantification of bouton complexity (perimeter/area) obtained from images like the middle image of (A); bouton complexity was unchanged in forskolin-treated terminals compared to controls (*p =* 0.27, unpaired *t* test). (D) Bar graph indicating the quantification of active zone density (AZ/μm^3^) obtained from 3D reconstructions like those in (B); AZ density was larger in forskolin-treated terminals (*p =* 0.0049, unpaired *t* test). (E) Bar graph indicating the quantification of presynaptic area (μm^2^) obtained from images like the middle image of (A); presynaptic area was unchanged in forskolin-treated terminals compared to controls (*p =* 0.07, unpaired *t* test). In all graphs, scatter points indicate individual boutons, *n =* 16 boutons for control and 14 boutons for forskolin-treated slices from 3 animals. Values represent mean ± SEM. The data underlying this figure can be found at doi: 10.5281/zenodo.4498214.
(TIF)

S6 Fig Synaptic vesicles disperse upon forskolin-induced presynaptic potentiation in chemically fixed acute slices. (A) Partial 3D reconstruction of hMFBs in control conditions (CTRL) or after forskolin treatment. Presynaptic membrane is green, postsynaptic membrane is light blue, and synaptic vesicles are blue (control) or red (forskolin). (B) Bar graphs indicating the quantification of synaptic vesicle density (SV/μm^3^); SV density was comparable in forskolin-treated and control terminals (*p =* 0.8629, unpaired *t* test). (C) Bar graphs indicating the quantification of synaptic vesicle distance from other synaptic vesicles normalized by the volume of the reconstruction (nm/μm^3^); distance between vesicles was increased in forskolin-treated terminals (*p =* 0.0186, Mann–Whitney *U* test). (D) Bar graphs indicating the quantification of mean nearest neighbor distance (MNND) between vesicles (nm); MNND was comparable in forskolin-treated and control terminals (*p =* 0.9136, Mann–Whitney *U* test). In all graphs, scatter points indicate individual boutons, *n =* 17 boutons for control and 14 boutons for forskolin-treated slices from 3 animals. Values represent mean ± SEM. The data underlying this figure can be found at doi: 10.5281/zenodo.4498214.
(TIF)

## Abbreviations

AAV: adeno-associated virus
ACSF: artificial cerebrospinal fluid
AZ: active zone
cAMP: cyclic adenosine monophosphate
EM: electron microscopy
EPSC: excitatory postsynaptic current
gSTED: time-gated stimulated emission depletion
hMFB: hippocampal mossy fiber bouton
HPF: high-pressure freezing
LTP: long-term potentiation
MNND: mean nearest neighbor distance
PB: phosphate buffer
PBS: phosphate buffer saline
PFA: paraformaldehyde
PPR: paired-pulse ratio
ROI: region of interest
RRP: readily releasable pool
STED: stimulated emission depletion
SV: synaptic vesicle
