# Supplementary figures and images for: Recruitment of release sites underlies chemical presynaptic potentiation at hippocampal mossy fiber boutons
Source: PLoS Biol. 2021 Jun 21;19(6):e3001149. doi: 10.1371/journal.pbio.3001149 (PMC8216508; doi:10.1371/journal.pbio.3001149)

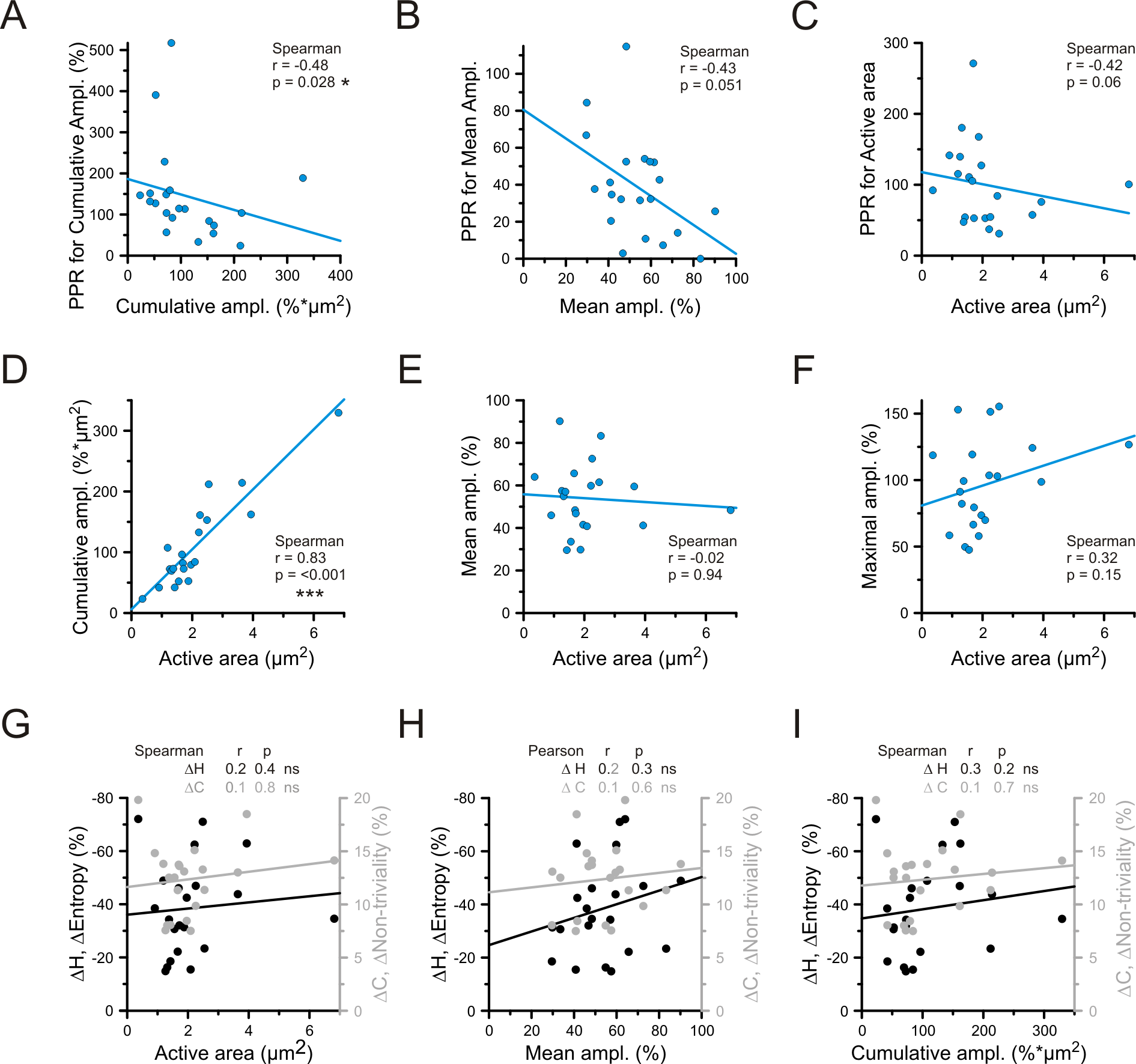

Supplement: S1 Fig — (A–C) Correlograms of cumulative amplitude (A), mean amplitude (B), and active area (C) versus their paired-pulse ratios demonstrate that among others cumulative amplitude (A) best reflects the activity-dependent form of short-term plasticity. (D–F) Correlograms of active area versus cumulative (D), mean (E), and maximal (F) amplitudes show that the active area is independent of glutamate concentration within the synaptic cleft (E and F), but is associated with the total amount of released glutamate (D), i.e., the measured active area reflects more a releasing area than a diffusional glutamate spread. (G–I) Correlograms of active area (G), mean amplitude (H), and cumulative amplitude (I) versus entropy and non-triviality change provide evidence that entropy and non-triviality are independent of the active area and amount of released glutamate. The data underlying this figure can be found at doi: 10.5281/zenodo.4498214. (TIF) [file pbio.3001149.s001.tif]

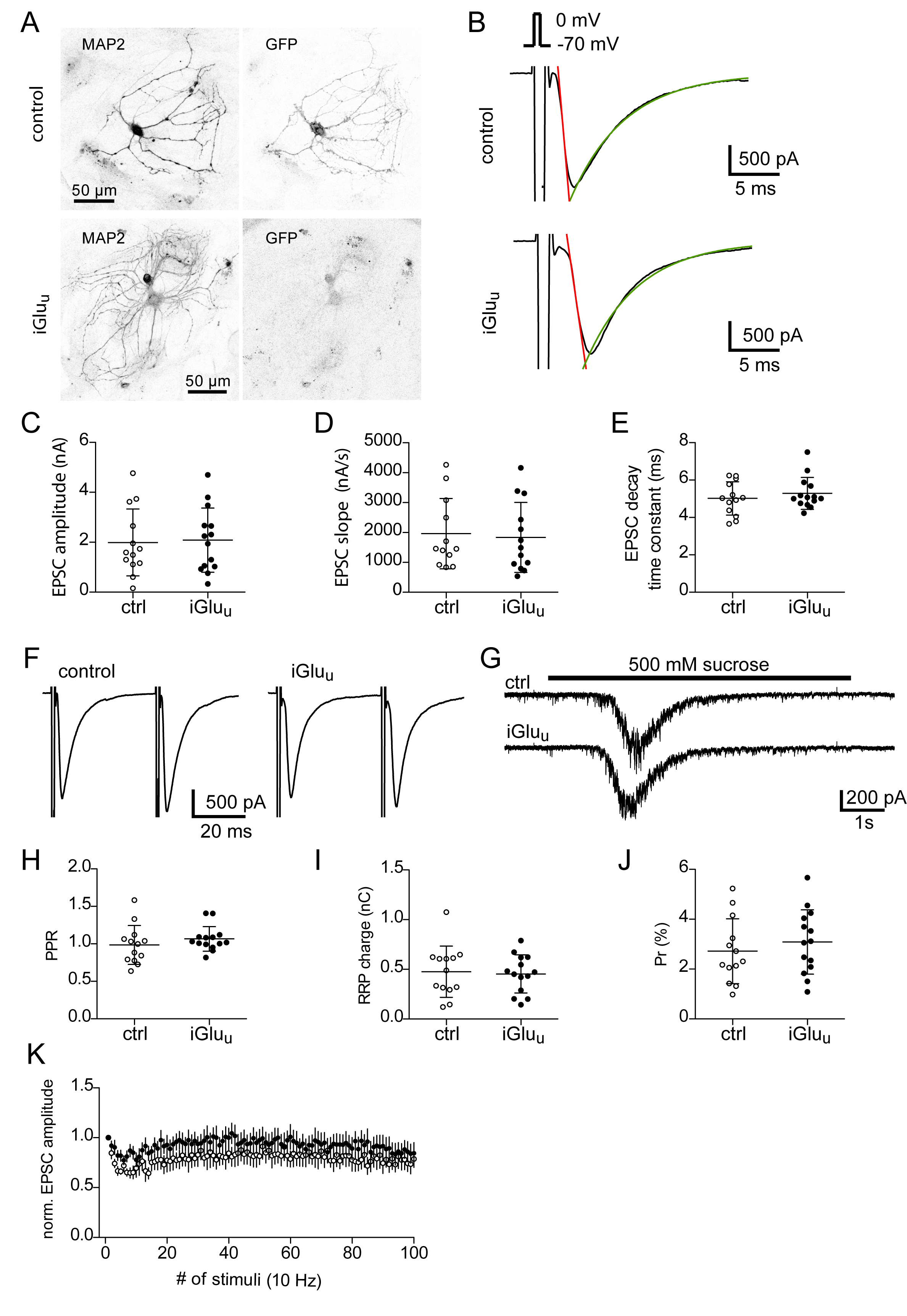

Supplement: S2 Fig — (A) Immunofluorescence images of cultured hippocampal neurons PFA-fixed at day in vitro 21 and stained for the dendritic marker MAP-2 and iGluu. The iGluu signal was enhanced by immunolabeling with anti-GFP antibodies. (B) Exemplary traces of whole-cell voltage clamp recordings of excitatory postsynaptic currents (EPSCs) evoked by 1-ms depolarizations in autaptic cultures of a noninfected control neuron, or neuron transduced with an AAV encoding iGluu. Fits for calculating the EPSC slope (red) and EPSC time constant (green) are shown as overlay. (C–E) EPSC amplitude (C), slope (D), and decay time constant (E) were not different between control and iGluu-expressing neurons. (F) Example traces of 2 EPSCs evoked at 25 Hz in a control and an iGluu-expressing neuron. (G) Typical synaptic currents evoked by brief applications of hypertonic sucrose solutions (500 mM) to deplete the readily releasable pool (RRP). (H–J) Paired-pulse ratio (PPR) (H), size of the RRP (I), and vesicular release probability (Pr) (J) were not significantly different between control and iGluu-expressing neurons. (K) Control neurons (open circles) and iGluu-expressing neurons (black circles) showed similar short-term kinetics of release during 10-Hz stimulation. Control: n = 12–13; iGluu: n = 12–14; N = 2 cultures for both conditions. The data underlying this figure can be found at doi: 10.5281/zenodo.4498214. (TIF) [file pbio.3001149.s002.tif]

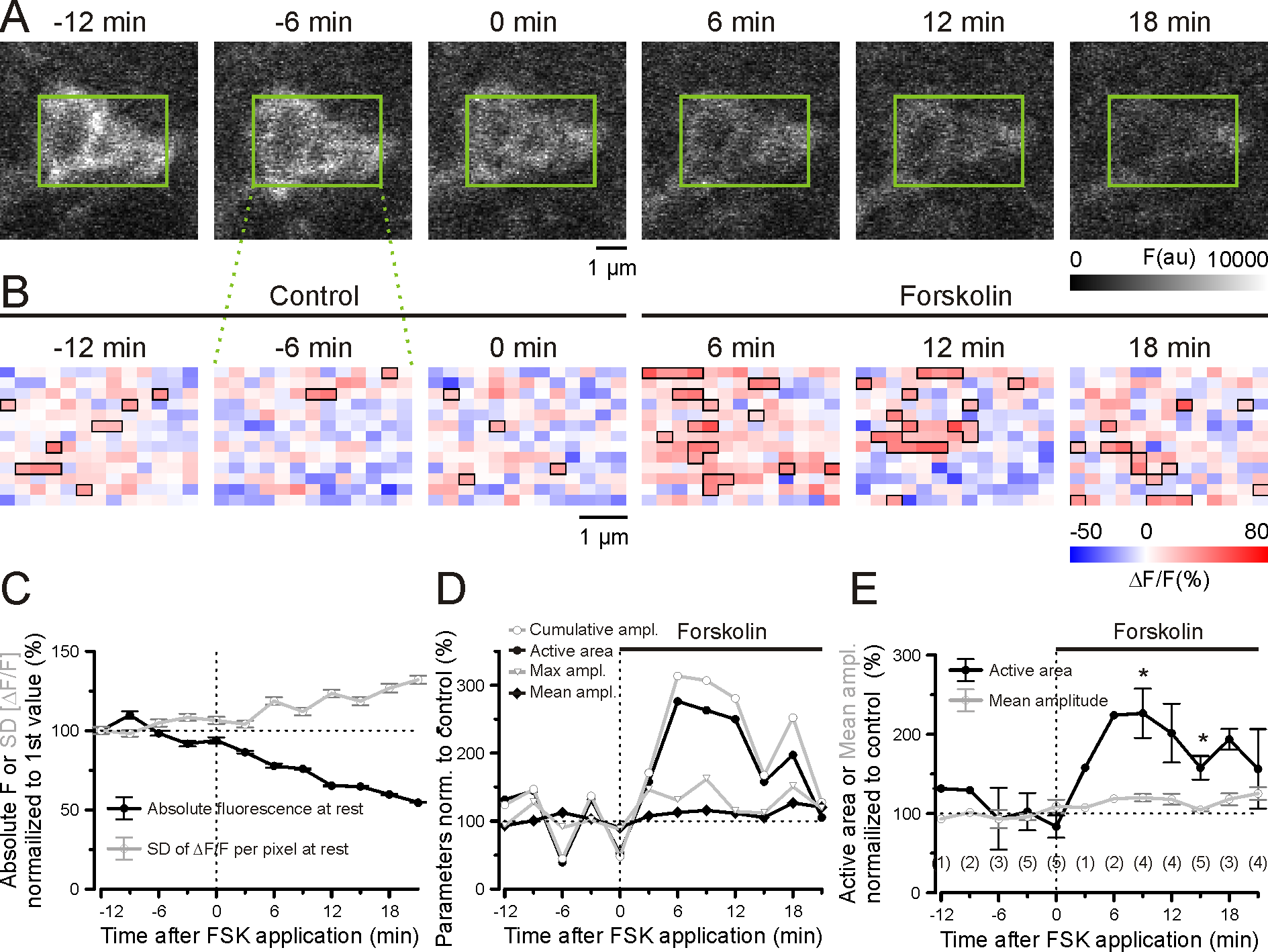

Supplement: S3 Fig — (A) Image sequence showing iGluu fluorescence for the hMFB recorded at different time points before (−12, −6, and 0 min) and after (6, 12, and 18 min) forskolin application. High-resolution images were taken before high-speed imaging. The green rectangle on the pictures marks the area of high-speed imaging. Note strong iGluu bleaching during recordings. (B) Sequence of pictures demonstrating ΔF/F spatial distributions at peak response to the first electrical stimulation acquired with high-speed imaging of marked (A) area for hMFB at different time points before (−12, −6, and 0 min) and after (6, 12, and 18 min) forskolin application. The contoured pixels represent the active area (pixels with response amplitude > 3 × SD of ΔF/F at rest). Note increased number of active pixels after forskolin application. (C) Graph demonstrating mean iGluu fluorescence signal F (black line and symbols) and mean standard deviation of ΔF/F at rest (gray line and symbols) for all pixels (n = 169) used for high-speed imaging. The data were calculated by averaging all values (mean F or SD of ΔF/F) calculated for each point of the baseline (50 ms prior to electrical stimulation). Data are normalized to the first value, 12 min before the forskolin application. Note 50% bleaching and 25% increased pixel noise. (D) Graph shows the cumulative amplitude, active area, and mean and maximal amplitudes of the iGluu fluorescent signal of the hMFB in (A and B). Data are normalized to the mean values of given parameters under control conditions. Note the forskolin-induced 200% increase in active area and cumulative amplitude, while mean and maximal amplitudes remain unaltered. (E) Graph demonstrating statistics for the active area (black line and symbols) and mean amplitudes (gray line and symbols) for 5 different hMFBs. Numbers in brackets show the number of data points used for averaging. Asterisks mark time points where the active area was significantly bigger than the active area at time point 0 m [file pbio.3001149.s003.tif]

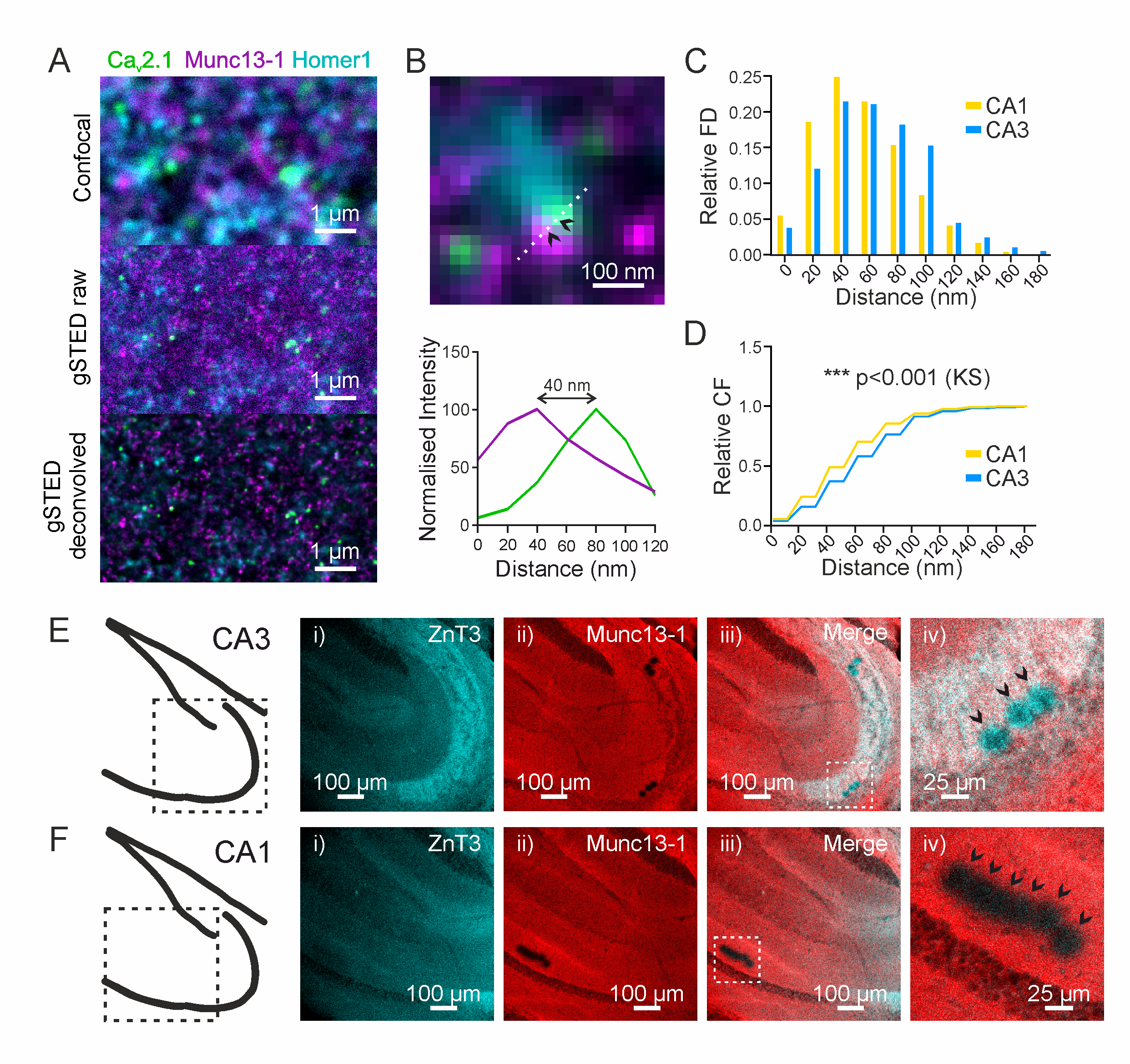

Supplement: S4 Fig — (A) Example scan in area CA1: confocal scan (top), raw gSTED scan (middle), and deconvolved gSTED scan (bottom). Staining for Cav2.1 (green), Munc13-1 (magenta), and Homer1 (cyan). (B) Example of an analyzed synapse in CA1: The distance between Cav2.1 (green) and Munc13-1 (magenta) was measured only if they were close to a Homer1-positive spot (cyan); line profiles were measured at the dotted line (top), drawn through the intensity maxima of the Cav2.1 and Munc13-1 signals (arrowheads). The distance was calculated between the intensity maxima of the Cav2.1 and Munc13-1 signals, shown in the corresponding normalized intensity plot (bottom). (C) Frequency distribution with a bin size of 20 nm for CA3 control (blue) and CA1 (yellow). The distribution of measured distances between Cav2.1 and Munc13-1 is different for CA1 compared to CA3 control. (D) Cumulative frequency with a bin size of 20 nm for CA3 control (blue) and CA1 (yellow). The cumulative distribution was significantly shifted to smaller values for CA1 compared to CA3 (p < 0.001, Kolmogorov–Smirnov test). (E) Imaging areas in CA3 were situated in the ZnT3-positive area. After STED imaging, images were taken at a confocal microscope to visualize the ZnT3 staining (i) and the imaging areas, which were bleached in the red channel by the second STED laser (ii) and situated within the ZnT3-positive mossy fiber band (iii) in the stratum lucidum, close to the CA3 pyramidal somata (iv). Imaging areas are indicated by black arrowheads. (F) Imaging areas in CA1. Confocal images were acquired after STED imaging to visualize the absence of ZnT3 in CA1 (i) as well as the bleached imaging areas in the red channel (ii), which were situated in area CA1 (iii) in stratum radiatum, with some distance to CA1 pyramidal somata (iv). Imaging areas are indicated by black arrowheads. The data underlying this figure can be found at doi: 10.5281/zenodo.4498214. (TIF) [file pbio.3001149.s004.tif]

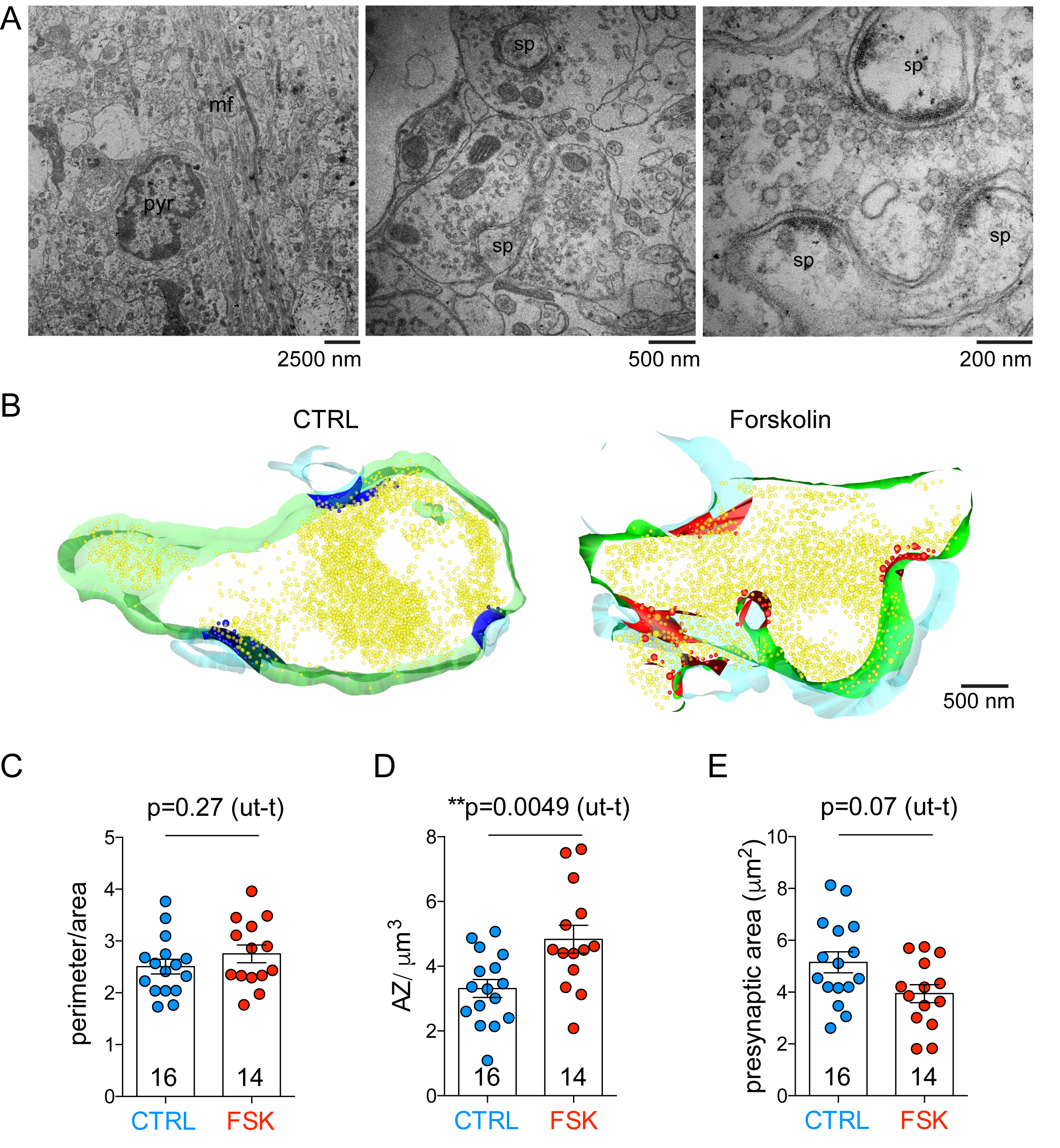

Supplement: S5 Fig — (A) Electron microscopy image of the stratum lucidum of the hippocampal CA3 region. A pyramidal cell soma (pyr) and mossy fiber axon bundles (mf) are visible in the left panel. In the central panel large presynaptic terminals contacting multiple spine heads (sp) are visible. The right panel shows a high-magnification image of 3 AZs. (B) Partial 3D reconstruction computed from manually segmented serial images of hMFBs in control conditions (CTRL) or after forskolin treatment. Presynaptic membrane is green, postsynaptic membrane is light blue, synaptic vesicles are yellow, and active zones and docked vesicles are blue (control) or red (forskolin). (C) Bar graph indicating the quantification of bouton complexity (perimeter/area) obtained from images like the middle image of (A); bouton complexity was unchanged in forskolin-treated terminals compared to controls (p = 0.27, unpaired t test). (D) Bar graph indicating the quantification of active zone density (AZ/μm3) obtained from 3D reconstructions like those in (B); AZ density was larger in forskolin-treated terminals (p = 0.0049, unpaired t test). (E) Bar graph indicating the quantification of presynaptic area (μm2) obtained from images like the middle image of (A); presynaptic area was unchanged in forskolin-treated terminals compared to controls (p = 0.07, unpaired t test). In all graphs, scatter points indicate individual boutons, n = 16 boutons for control and 14 boutons for forskolin-treated slices from 3 animals. Values represent mean ± SEM. The data underlying this figure can be found at doi: 10.5281/zenodo.4498214. (TIF) [file pbio.3001149.s005.tif]

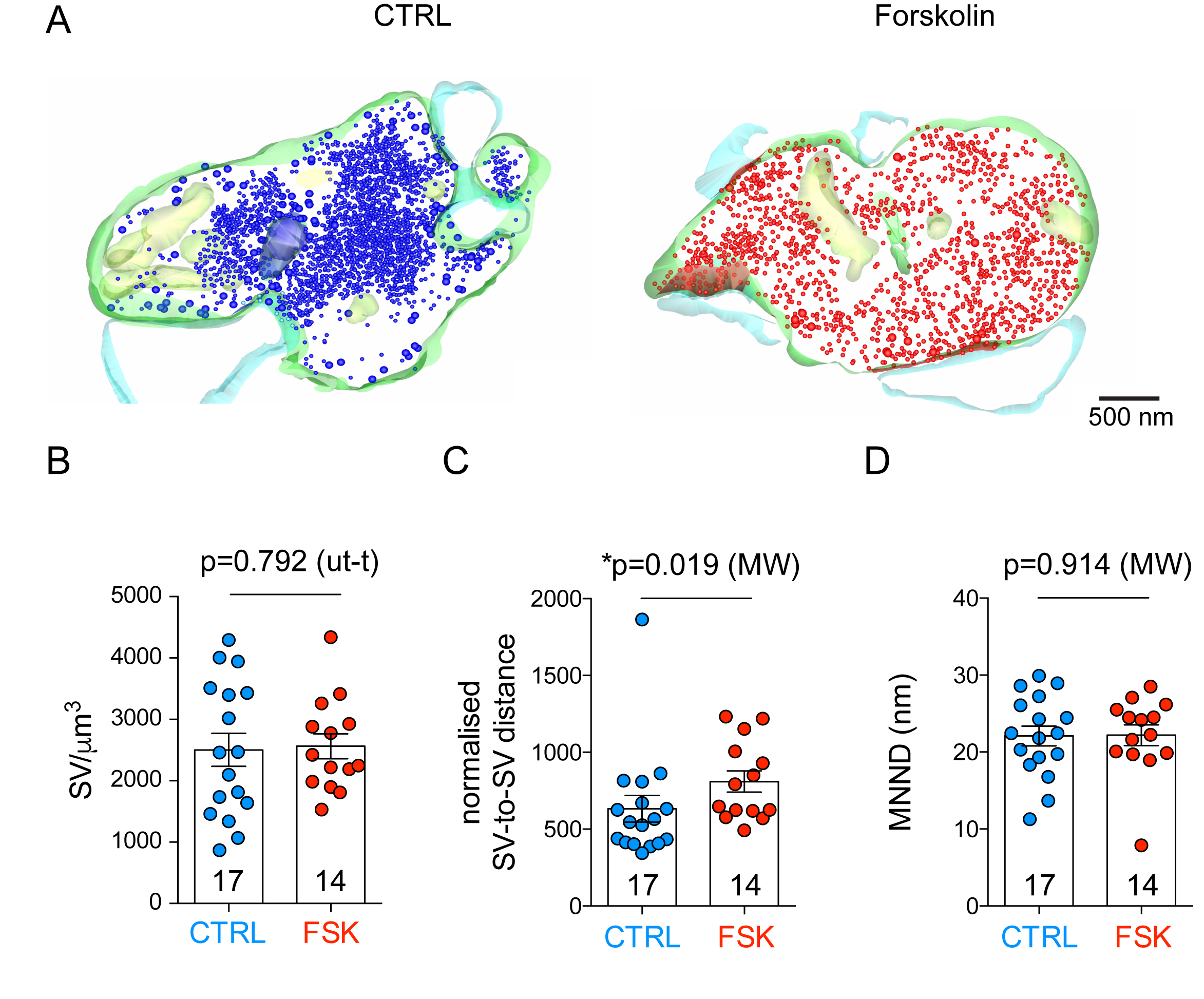

Supplement: S6 Fig — (A) Partial 3D reconstruction of hMFBs in control conditions (CTRL) or after forskolin treatment. Presynaptic membrane is green, postsynaptic membrane is light blue, and synaptic vesicles are blue (control) or red (forskolin). (B) Bar graphs indicating the quantification of synaptic vesicle density (SV/μm3); SV density was comparable in forskolin-treated and control terminals (p = 0.8629, unpaired t test). (C) Bar graphs indicating the quantification of synaptic vesicle distance from other synaptic vesicles normalized by the volume of the reconstruction (nm/μm3); distance between vesicles was increased in forskolin-treated terminals (p = 0.0186, Mann–Whitney U test). (D) Bar graphs indicating the quantification of mean nearest neighbor distance (MNND) between vesicles (nm); MNND was comparable in forskolin-treated and control terminals (p = 0.9136, Mann–Whitney U test). In all graphs, scatter points indicate individual boutons, n = 17 boutons for control and 14 boutons for forskolin-treated slices from 3 animals. Values represent mean ± SEM. The data underlying this figure can be found at doi: 10.5281/zenodo.4498214. (TIF) [file pbio.3001149.s006.tif]
